# Oversampling and replacement strategies in propensity score matching: a critical review focused on small sample size in clinical settings

**DOI:** 10.1186/s12874-021-01454-z

**Published:** 2021-11-22

**Authors:** Daniele Bottigliengo, Ileana Baldi, Corrado Lanera, Giulia Lorenzoni, Jonida Bejko, Tomaso Bottio, Vincenzo Tarzia, Massimiliano Carrozzini, Gino Gerosa, Paola Berchialla, Dario Gregori

**Affiliations:** 1 Unit of Biostatistics, Epidemiology and Public Health, Department of Cardiac, Thoracic, Vascular Sciences and Public Health, University of Padova, Via Loredan 18, 35121 Padova, Italy; 2 Department of Cardiac, Thoracic,Vascular Sciences and Public Health, University of Padova, Padova, Italy; 3 Department of Clinical and Biological Sciences, University of Torino, Torino, Italy

**Keywords:** Propensity score matching, Oversampling, Replacement, Small samples, Monte Carlo simulations

## Abstract

**Background:**

Propensity score matching is a statistical method that is often used to make inferences on the treatment effects in observational studies. In recent years, there has been widespread use of the technique in the cardiothoracic surgery literature to evaluate to potential benefits of new surgical therapies or procedures. However, the small sample size and the strong dependence of the treatment assignment on the baseline covariates that often characterize these studies make such an evaluation challenging from a statistical point of view. In such settings, the use of propensity score matching in combination with oversampling and replacement may provide a solution to these issues by increasing the initial sample size of the study and thus improving the statistical power that is needed to detect the effect of interest. In this study, we review the use of propensity score matching in combination with oversampling and replacement in small sample size settings.

**Methods:**

We performed a series of Monte Carlo simulations to evaluate how the sample size, the proportion of treated, and the assignment mechanism affect the performances of the proposed approaches. We assessed the performances with overall balance, relative bias, root mean squared error and nominal coverage. Moreover, we illustrate the methods using a real case study from the cardiac surgery literature.

**Results:**

Matching without replacement produced estimates with lower bias and better nominal coverage than matching with replacement when 1:1 matching was considered. In contrast to that, matching with replacement showed better balance, relative bias, and root mean squared error than matching without replacement for increasing levels of oversampling. The best nominal coverage was obtained by using the estimator that accounts for uncertainty in the matching procedure on sets of units obtained after matching with replacement.

**Conclusions:**

The use of replacement provides the most reliable treatment effect estimates and that no more than 1 or 2 units from the control group should be matched to each treated observation. Moreover, the variance estimator that accounts for the uncertainty in the matching procedure should be used to estimate the treatment effect.

**Supplementary Information:**

The online version contains supplementary material available at 10.1186/s12874-021-01454-z.

## Introduction

Inferences on the effects of treatments or exposures are increasingly found by using observational studies [1]. In such situations, the lack of randomization does not ensure the overall balance of individual baseline characteristics. Thus, statistical methods that can detect the treatment effect on an outcome of interest while controlling for potential confounders are needed. Propensity score (PS) methods are among the most used approaches in the medical literature for addressing the impacts of therapies or exposures. In particular, the use of propensity score matching (PSM) is widespread in clinical studies because of its ability to mimic a randomized clinical trial (RCT) in which the effect of a therapy is evaluated by comparing the outcomes of treated and control subjects belonging to the matched sample [1].

PSM methods have become very popular in cardiothoracic surgery [2–6], especially when the goal is to evaluate a new therapy or a new surgical procedure and compare it to the current standard approaches. In these settings, two main issues hamper the inference process: the selection bias and the small sample size. The former arises because performing a randomized study in this situation is commonly not ethically acceptable. The latter is of greater concern since the small number of subjects significantly undermines the statistical power that is needed to detect a clinical effect. A low number of individuals is a common feature of cardiothoracic surgery studies as sample sizes often range from less than 100 subjects to a few hundred subjects [7–10]. Constructing a matched sample using standard PSM methods, such as 1:1 matching without replacement, could further reduce the initial sample size of the study, thus leading to an inaccurate comparison of different surgical procedures among matched subjects. Some studies addressed this issue using PSM with oversampling, i.e., matching more than one control to each treated variable or more than one treated variable to each control [11, 12]. The main idea behind this approach is to create a matched set of individuals with a larger size than the one that would be obtained using classical 1:1 matching to increase the statistical power that is needed to detect the potential effect of interest. Furthermore, the use of matching with replacement may be useful for finding all the matched units from the control group, which is defined by the level of oversampling, e.g., 5 control units.

To the best of our knowledge, no previous study evaluated the performances of the combination of replacement and oversampling in PSM. This study aims to assess whether performing PSM with replacement and oversampling can eventually address the problem of small sample size and result in valid inference for treatment effect estimation. We carried out the investigation using Monte Carlo simulations, and we applied the proposed approaches to a real case study. As a motivating example, we use the data from the study of Bejko et al. (2018) [13], in which the outcomes of different continuous-flow left ventricular assist devices are compared. The remainder of this paper is organized as follows. In Sect. 3, we briefly introduce the PSM framework and describe PSM with replacement and oversampling. In Sect. 4, we describe the Monte Carlo simulations that are used to evaluate the performance of PSM with and without replacement for different levels of oversampling. In Sect. 6, we present the analysis of the case study. In Sect. 7, we summarize the findings and provide some recommendations for the implementation of the method.

## Methods

### PSM framework

The potential outcomes framework was proposed by Rubin (1974) [14]. We use $$i=1,\ldots\ ,n$$ to represent the $$i-th$$ subject of the *N* subjects that are enrolled in a study. When evaluating the effect of a binary treatment, one individual has two potential outcomes as follows: $${Y}_{i}(0) $$ and $${Y}_{i}\left(1\right)$$. The former denotes the outcome that is observed if the subject is assigned to the control group, while the latter is the outcome that is observed if the subject is assigned to the treatment group. Let $$T$$ be an indicator of the binary treatment that denotes the actual group to whom the individual had been assigned ($$T=0$$ if the subject is assigned to the control group and $$T=1$$ if the subject is assigned to the treatment group). The fundamental problem of causal inference lies in the fact that only one of the two outcomes can be observed for each subject, i.e., the outcome under the actual group of treatment, which is defined as $${\mathrm{Y}}_{\mathrm{i}}={\mathrm{T}}_{\mathrm{i}}{\mathrm{Y}}_{\mathrm{i}}(1)+\left(1-{T}_{\mathrm{i}}\right){\mathrm{Y}}_{\mathrm{i}}(0)$$. In such situations, the impact of a treatment can be evaluated using the average treatment effect (ATE), which is defined as the average difference between individual potential outcomes:

$$E\left[{Y}_{i}(1) -{Y}_{i}(0)\right]$$

and the average treatment effect on the treated (ATT), which is the average difference between individual potential outcomes for subjects who had been assigned to the treatment group:

$$\text{E}\left[{Y}_{i}(1) -{Y}_{i}(0)|T=1\right]$$

PS is defined as the individual probability of being assigned to the treatment group given the baseline characteristics of the subject, i.e., $P\left(T=1|X\right)$, where $X$ is a set of baseline characteristics. As demonstrated in the seminal PS paper [15], the distributions of the pre-treatment variables between treated and control subjects are similar when conditioning on the PS only if two conditions are satisfied: (i) no unmeasured confounding, i.e. $Y (1) , Y (0) \perp T |X$ and (ii) positivity assumptions, i.e. $0<P\left(T=1|X\right)<1$.

PSM is a PS method used to remove residual confounding when estimating treatment effect by forming matched sets of treated and control units with similar values of PS. The effect of a treatment can be then assessed by comparing the outcomes of the treated and control subjects included in the matched set, mimicking the standard statistical analysis of an RCT.

Several matching algorithms have been proposed when matching on the PS: pair matching, many-to-one matching, full matching, nearest neighbour (NN) matching, matching with a calliper, optimal matching, matching with replacement, and matching without replacement [16–20]. The most classical implementation of PSM is 1:1 NN matching without replacement, which can be performed with or without imposing a calliper. Each treated subject is paired with one control subject, and the matched control from the control “reservoir” is discarded. The ideal situations for 1:1 matching without replacement are those when each treated individual received a match. Indeed, such a matching process is easier to implement when a large pool of controls is present in the study. The matched set formed with this approach allows the analyst to estimate the ATT when all the units from the treatment group receive a matched unit from the control group [21]. If some of the treated cannot be matched then the estimand of interest may not generalize to the target population defined by design. Matching with replacement represents a valuable approach when not all the treated are matched to control units. Indeed, if control subjects are used as candidate matching multiple times, each treated unit can be matched to a control unit.

### PSM with replacement and oversampling

Classical 1:1 matching without replacement may not be the most suitable approach when the number of individuals enrolled in a study is small, and when an imbalance in terms of treated and untreated subjects is present. Moreover, the initial sample size of the study, which is generally small, can be further reduced, increasing the sampling variability associated with the treatment effect estimate and reducing the accuracy of the findings.

In the present paper, we propose the use of different PSM methods to face the issues that may be encountered using the classical PSM approach in clinical studies characterized by small sample sizes and a high imbalance in the distributions of pre-treatment covariates. More specifically, we evaluate if the combination of matching with replacement and oversampling (1:K matching, where K > 1) can aid in increasing precision and accuracy of causal estimates. By matching treated subjects with more than one control with replacement, one can potentially ensure that all the treated units receive a matched control.

## Monte Carlo simulations

The performances of PSM with different combinations of replacement and oversampling were compared using Monte Carlo simulations. We considered the setting with a binary outcome and a binary treatment for two reasons. First, it is the most common setting in the medical literature and, second, it mimics the situation of our motivating example in which a dichotomous clinical outcome is compared between subjects undergoing two different surgical procedures. Planification and description of Monte Carlo simulations were structured using the aims, data-generating mechanisms, estimands, and performance measures (ADEMP) guidelines [22].

### Aims

The aim of the Monte Carlo simulations is to compare PSM strategies with replacement and oversampling in terms of (1) overall balance of baseline covariates in the matched sample, (2) bias of the ATT estimator and (3) coverage of 95 % Confidence Intervals (CIs).

### Estimand

The ATT was evaluated in terms of absolute risk reduction, a measure that is argued to be of greater importance for clinical decision making than relative measures such as relative risks and odds ratios [23, 24] when binary outcomes are considered.

### Data-generating mechanism

In each dataset, we simulated 6 baseline covariates, which we represent as $X=\left({X}_{1},{X}_{2},\ldots ,{X}_{6}\right)$, from a latent multivariate normal distribution, setting means equal to 0. The covariance matrix was derived from a correlation matrix with all diagonal elements equal to 1 (which implies a standard deviation of 1 for all the variables) and the non-diagonal elements with values ranging from 0.1 to 0.5. Indeed, we considered a scenario with different degrees of dependence among baseline covariates, which can often be encountered in practice. The first three covariates were transformed into binary variables by choosing the cutoff values such that the marginal probabilities were approximately equal to 0.25, 0.3, and 0.2, respectively.

For each subject, we computed the probability of treatment assignment using a logistic model as follows:

$$logit\left({p}_{{T}_{i}}\right)={\beta }_{0}+{\beta }_{1}{x}_{i1}+{\beta }_{2}{x}_{i2}+{\beta }_{3}{x}_{i3}+{\beta }_{4}{x}_{i4}+{\beta }_{5}{x}_{i5}+{\beta }_{6}{x}_{i6}$$

where ${p}_{{T}_{i}}$ denotes the probability of treatment selection. We considered two situations where the baseline covariates have different impacts on the treatment assignment mechanism. The individual probabilities of being assigned to the treatment group are governed by the vector of the treatment model coefficients, i.e., $\tilde{\beta}=\left(\beta_1,\beta_2,\ldots,\beta_6\right)$. In the first situation, which we will denote as the "weak" treatment assignment, $\tilde{\beta}$ was set equal to $\left(\log(1.25) ,\log(1.5) ,\log(1.25) ,\log(1.5) ,\log(1.25) ,\log(1.5) \right)$. In the second situation, which we will denote as the “strong” treatment assignment, $\tilde{\beta}$ was set equal to $\left(\log(1.5) ,\log(1.75) ,\log(1.5) ,\log(1.75) ,\log(1.5) ,\log(1.75) \right)$. The value of the intercept ${\beta }_{0}$ of the treatment model was imposed such that the proportions of treated subjects were equal to 0.3, 0.5 and 0.7 using a grid search approach (values are shown in Supplementary Table S11). Each subject treatment status $T$ was generated from a Bernoulli distribution with an individual probability that was computed from the treatment model.

For the main set of simulations, a binary outcome *Y* was generated from a Bernoulli distribution with a subject-specific probability ${p}_{{Y}_{i}}$ that was computed using a logistic model as follows:

$$logit\left({p}_{{Y}_{i}}\right)={\gamma }_{0}+{\gamma }_{1}{x}_{i1}+{\gamma }_{2}{x}_{i2}+{\gamma }_{3}{x}_{i3}+{\gamma }_{4}{x}_{i4}+{\gamma }_{5}{x}_{i5}+{\gamma }_{6}{x}_{i6}+{\gamma }_{\text{T}}T$$

The vector of the outcome model coefficients $\tilde{\gamma}=\left(\gamma_1,\gamma_2,\ldots,\gamma_6\right)$ associated with the baseline covariates was fixed using the following values: $\left(\log (1.25) ,\log(1.25) ,\log(1.5) ,\log(1.5) ,\log(1.75) ,\log(1.75) \right)$. By using this model, we fixed the baseline covariates at different levels of confounding with characteristics that act both as weak and strong confounders, which is very common in the medical literature. We selected the value of the outcome model intercept ${\gamma }_{0}$ such that the occurrence of the outcome $Y$ was equal to 0.20 using the same approach that was employed for ${\beta }_{0}$ (values are shown in Supplementary Table S11). The value of the parameter ${\gamma }_{\text{T}}$was chosen such that the ATT, measured as absolute risk reduction, was equal to approximately 0.15, using a data-generating method for binary outcomes in which the treatment produces a specified risk difference (values are shown in Supplementary Table S11) [25]. The data-generating algorithm exploits the fact that the absolute risk reduction is a collapsible measure, i.e. the marginal or population absolute risk reduction equals the average subject-specific absolute risk reduction [26].

Finally, we allowed the sample size to vary to evaluate how different PSM combinations of replacement and oversampling act in different sample size situations. We considered the four different settings as follows: 100 subjects, 250 subjects, 500 subjects, and 1000 subjects. The situations that are of interest in this study are those with limited numbers of subjects (100 and 250), as in our motivating example.

In the main set of simulations, the choice of simulating the binary outcome from a logistic regression model was based on several considerations. First, the logit function allows any values of the linear predictor on the whole real line to be transformed into a valid expected probability constrained between 0 and 1. Second, logistic regression is widely used in medical settings where the interest is to relate the expected probabilities of a clinical event given a set of covariates. Thus, being the most popular model for medical binary data, several simulation studies used it as starting point when evaluating dichotomous outcomes. Third, logistic regression allows expressing the effects of covariates both on the relative scale, e.g. odds ratios, and absolute scale, risk difference, which can be of great relevance for clinical interpretation and decision-making [27, 28]. Given that the estimand of interest is expressed on the difference scale, the use of the logit transformation induced heterogeneity in the treatment effect. Despite this is not necessarily a concern and it might also reflect more realistic clinical situations, we performed a second round of simulations by simulating the outcome from a linear probability model, which assumes that the treatment effect is additive and linear on the probability scale, to evaluate the statistical properties of the proposed estimators both in presence and absence of treatment effect heterogeneity. The parameters used to set up the second round of simulations are reported in Supplementary Table S16.

For both the primary and secondary sets of simulations, we considered a total of 24 scenarios, which included all the possible combinations of the treatment assignment mechanisms, the proportions of treated subjects, and the sample sizes. For each scenario, we drew 10,000 datasets on which the statistical analysis was performed.

### Methods

For each simulated dataset, the individual PSs were estimated using a logistic model that included all the covariates as main effects. Matching was then performed using the NN algorithm with a calliper set to 0.2 of the standard deviation of the logit of the PS distribution, a value that was shown to result in good performances in several settings [29]. Within every simulated dataset, we ran a total of 10 matching strategies over all the combinations of replacement (with and without replacement) and levels of oversampling, which we defined by varying the number of matched controls K from 1 to a maximum of 5. The balance of the distributions of the covariates in the matched set was assessed using the average standardized mean difference (ASMD) and the overlapping coefficient (OVL), two measures that quantify the degree of imbalance for all the covariates simultaneously [30, 31]. The ASMD is the mean value of the standardized mean differences (SMDs) of the covariates between the compared groups. The OVL corresponds to the proportion of overlap in the density functions of the PS estimated in treatment and control groups on the matched set. It ranges between 0 and 1, and the higher the value, the higher the balance. We used 1-OVL to make it comparable with ASMD. Moreover, the goodness of the matching set of observations was also assessed by computing the Proportion of Matched Treated (PMT). A PMT closer to 1 indicates that most of the units from the treatment group are retained in the matched set, i.e. the estimated ATT is still generalizable to the target population of treated subjects. For each matched set of subjects, we evaluated the impact of the treatment by estimating the ATT as the weighted absolute risk reduction of the treatment. Each paired set of observations was assigned a weight equal to the reciprocal of times the control unit was matched to a treated unit [32–34]. CIs at the 95 % level were computed using a method that accounts for the matched nature of the sample used to estimate the ATT [35, 36]. Moreover, we also evaluated the 95 % CIs obtained with the Abadie-Imbens (AI) method, which accounts for the uncertainty associated with the matching procedure [37]. We will refer to the methods as “standard” and “AI”, respectively.

### Performance measures

The performances of overall balance were assessed by averaging ASMD, OVL and PMT values over the 10,000 datasets simulated in each scenario. The performances of the ATT estimator on the set of observations obtained using PSM with replacement and oversampling were evaluated using the following three criteria: the relative bias, the root mean squared error (RMSE), and the nominal coverage (NC). Bias is defined as the distance between the estimated and the true ATT, i.e., $bias=\overline{\hat{ATT}}-ATT_{true}$, where $\hat{ATT}$ is the average estimated ATT across all the simulated datasets. To make it comparable across the scenarios, we considered a relative version of the bias, computed as $\left|bias/{ATT}_{true}\right|*100$, which ranges between 0 and 100, with higher values indicating higher bias. RMSE is defined as the square root of the sum of the variance of the ATT estimates across the simulated datasets and the square of the bias, i.e., $RMSE=\sqrt{V\left(\hat{ATT}\right)+bias^2}$. RMSE is a measure that combines information from both the bias and the sampling variance associated with the ATT estimate. Lower RMSEs denote better estimators of the ATT. Finally, NC denotes the percentage of times that the 95 % CI includes the true ATT across all the simulated datasets. If the method is valid, then the NC will be close to 0.95.

All the analyses were conducted using the R statistical programming language (version 4.0.2) [38]. The R package *Matching* (version 4.9-7) was used to construct the matched samples, estimate the ATT, and the relative standard error [39]. The balance in the matched samples was evaluated using the *cobalt* R package (version 4.2.2) [40]. The R code for reproducing the results of the simulations is available on Github (https://github.com/UBESP-DCTV/psm.oversampling).

### Simulation results

**Fig. 1:**
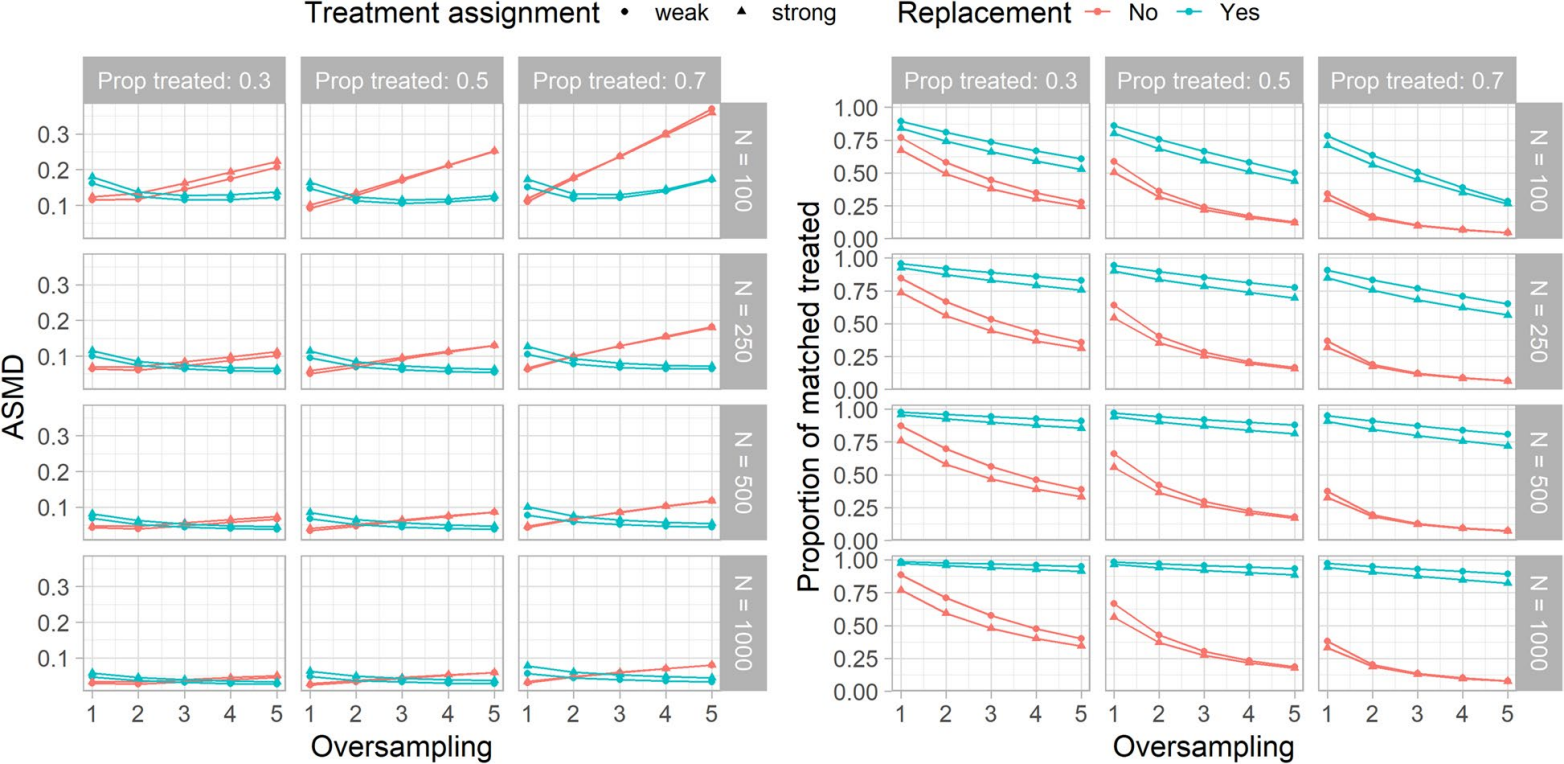
Balance assessment on the matched sets obtained in each scenario of the Monte Carlo simulations. The plot on the right shows the Average Standardized Mean Differences (ASMDs), and the left-graph the proportion of matched treated. On the x-axis, the level of oversampling is represented. Matching without and with replacement are identified by the colors. The shape of the dots distinguishes between weak and strong treatment assignment. The columns of the panel grids show the proportion of treated subjects in the dataset, whereas the rows show the sample size of the dataset

We first evaluate the goodness of the PSM strategies in terms of the balance of the baseline characteristics in the matched sets, using the ASMDs and the OVL, and the PMT. Since the OVL results were in line with the ASMDs, only the latter are presented. All the measures were averaged over the 10,000 simulated datasets. Figure 1 shows the ASMDs and the PMT obtained across all the scenarios considered in the Monte Carlo simulations. In all the scenarios, matching without replacement showed a better balance than matching with replacement when classical 1:1 matching was performed. For higher levels of oversampling (more than one control unit matched to each treated), the opposite relationship was observed. More generally, the overall balance was better for increasing levels of oversampling when matching was performed with replacement than when matching was done without replacement. Regarding the PMT, matching with replacement discarded fewer observations from the treatment group than matching without replacement. Moreover, the PMT decreased for increasing levels of oversampling, both when matching was performed without and with replacement since not all the treated units received the prespecified number of control units. The decrease was less pronounced for larger sample sizes (500 and 1000 units) when matching was performed with replacement. Negligible differences were observed between weak and strong treatment assignment scenarios for both ASMDs and PMT.

**Fig. 2:**
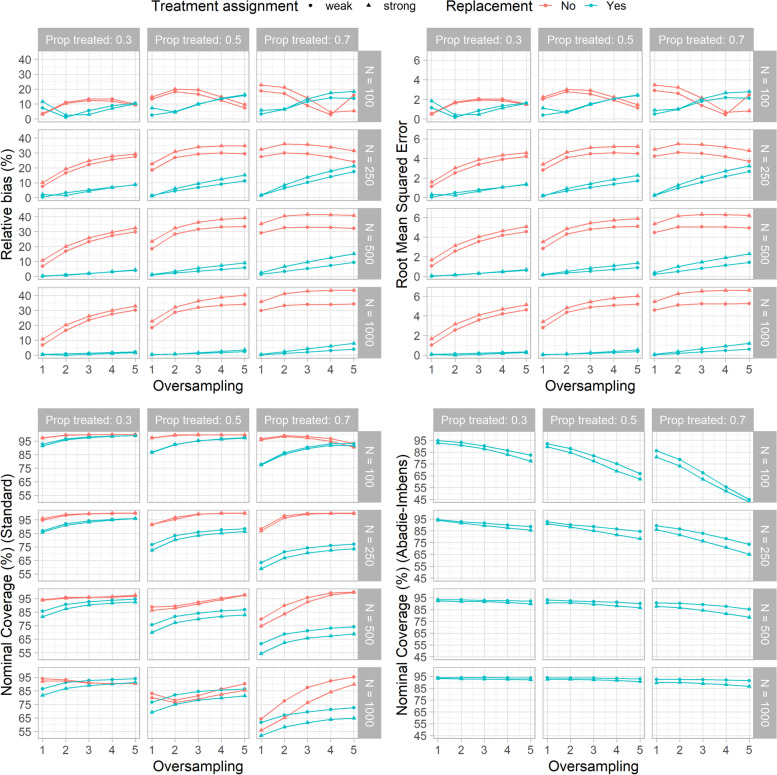
Performances of the Average Treatment effect on the Treated (ATT) estimator on the matched sets obtained in each scenario of the primary set of Monte Carlo simulations. The top-left plot shows the relative bias, whereas the Root Mean Squared Error (RMSE) is shown in the top-right plot. On the bottom-left side, the 95 % Nominal Coverage (NC) obtained with the standard method is depicted, whereas on the bottom-right side the 95 % NC obtained with the Abadie-Imbens (AI) method is shown. On the x-axis, the level of oversampling is represented. Matching without and with replacement are identified by the colors. The shape of the dots distinguishes between weak and strong treatment assignment. The columns of the panel grids show the proportion of treated subjects in the dataset, whereas the rows show the sample size of the dataset

The relative bias, the RMSE, and the 95 % NC results for the main simulations are depicted in Fig. 2 (raw numbers are show in Supplementary Table S12-S15). In almost all the scenarios, matching with replacement produced less biased ATT estimates than matching without replacement. The relative bias increased for higher levels of oversampling in all the scenarios except when matching was performed without replacement in settings with a low sample size (n=100) and 0.7 of subjects were assigned to the treatment group. The pattern was less evident for higher sample sizes (500 and 1000 units) when matching was done with replacement, with a relative bias always under 10 %. Higher bias values were observed when the treatment assignment was strong, as expected. Regarding the RMSE, the results are in line with those observed for the relative.

bias: higher RMSE values were observed when matching was done without replacement and for higher levels of oversampling in almost all the scenarios.

The results observed for the 95 % NC obtained with the standard method (bottom-left plot of Fig. 2) were somewhat in contrast with those observed for relative bias and RMSE. Overall, the 95 % CIs obtained when matching was performed with replacement were always lower than when matching was done without replacement. These differences were more apparent for higher proportions of treated subjects, especially when it was equal to 0.7. In most of the scenarios, the estimates obtained with matching without replacement were closer to the nominal coverage except when the sample size was high (500 and 1000 units) and when the proportion of treated was 0.5 and 0.7. When oversampling was used, improved coverage was reached up to 2-3 control units matched to each treated subject. No improvement was observed for higher levels of oversampling. Overall, the NC was highly influenced by the proportions of treated subjects and the sample size.

The bottom-right plot of Fig. 2 shows the 95 % NC obtained with the AI method. The method was applied only when matching was done with replacement. Higher levels of oversampling were associated to lower 95 % NC, especially in low sample sizes (100 and 250 observations) and higher proportions of treated subjects settings. In contrast to that, the difference was negligible when the sample size was higher (500 and 1000 units), with coverage often close to the nominal value. Overall, the AI method produced more accurate coverage than the standard method when matching was performed with replacement.

 The results of the relative bias, the RMSE, and the 95 % NC from the second run of simulations are reported in Supplementary Figure S1 and Supplementary Table S17-S20. Regarding the relative bias, findings were similar to those observed in main simulations for a sample size of 100 units, except that matching without replacement produced more biased estimates for higher oversampling levels than the primary simulations. However, for higher sample sizes, the bias of the matching methods was nearly null in all the scenarios. Results from RMSE were equivalent to those from bias, as also observed in the primary simulations. For 95 % NC, correct coverages were observed in almost all the scenarios when 1:1 matching without replacement was used. However, for increasing levels of oversampling the coverage was always higher than the nominal level, suggesting that the estimator of the standard error was too conservative. When replacement was considered, the coverage was always below the nominal level in all the scenarios but when the proportion of treated subjects was 0.3 and the matching ratio was at least 3 or 4. When the AI estimator for standard errors was used, the coverage improved and the results were in line with the primary simulations.

### Case study

#### Data sources

The study aimed to compare two different left ventricular assist devices (LVADs) that were implanted during recent years at the cardio surgery “V. Gallucci” center at the University of Padova in Italy: the Jarvik 2000 LVAD (Jarvik Heart, Inc., New York, USA) and the Heartware HVAD (HeartWare, Inc., Framingham, MA). LVADs are used to treat patients with end-stage heart failure that is nonresponsive to medical and conventional surgical therapy. The endpoints of the study were the in-hospital mortality, long term survival, all causes of death, early and late driveline infections (DLI), acute post-LVAD, and chronic right ventricular failure (RVF), and antithrombotic-therapy related complications. In our study, we focused our attention on the occurrence of both early and late DLI.

The data on the patient demographics, medical history, analysis tests, and clinical characteristics were collected for this sample. The descriptive statistics of the sample are reported in Table 1. The differences in the variable distributions by LVAD groups are reported in terms of the SMDs. Several variables were highly different from one treatment group to the other, thereby denoting that the assignment to the device group was highly dependent on individual baseline characteristics. Overall, 103 patients were enrolled in the study, of which 46 (44.5\%) were treated with the HeartWare HVAD and 57 (55.5\%) were treated with the Jarvik 2000 LVAD.

### Statistical analysis

Among the 36 baseline covariates, we selected the following: *age_at_implant (age of the patient at the time of the LVAD implant treatment)*, *BSA (body surface area), days_cvvh_preo >1 (if the patient was treated with Continuous Veno-Venous Hemofiltration in the preoperative period due to renal insufficiency for more than 1 day)*, *EF (ejection fraction)*, *INTERMACS IV profiles (if the patient was assigned to the fourth level of the Interagency Registry for Mechanically Assisted Circulatory Support scale)* and *REDO (reintervention)*. We chose these 6 baseline covariates following the suggestion of Brookhart et al. (2006) [41], which was that including only those.

**Table 1:** Descriptive statistics of the case study sample stratified by LVAD groups. Continuous variables are represented with I quartile/median/III quartile and categorical variables with percentage (relative frequencies). The Standardized Mean Differences (SMDs) on the unbalanced case study sample are reported in the last column of the table

	*Combined (N=103)*	*N*	*HeartWare HVAD (N=46)*	*Jarvik2000 LVAD (N=57)*	*SMD*
Sex (Female)	15 % (15)	103	17 % ( 8)	12 % ( 7)	-0.14
Intermacs I	42 % (43)	103	39 % (18)	44 % (25)	0.10
Intermacs II	27 % (28)	103	39 % (18)	18 % (10)	-0.49
Intermacs III	15 % (15)	103	13 % ( 6)	16 % ( 9)	0.08
Intermacs IV	17 % (17)	103	9 % ( 4)	23 % (13)	0.39
Age (years)	50/60/66	103	42/53/62	58/63/67	0.89
BSA	1.8/1.9/2.0	103	1.7/1.8/2.0	1.8/1.9/2.0	0.48
Cardiomyopathy : DCM	38 % (39)	103	52 % (24)	26 % (15)	-0.55
IHD	55 % (57)		43 % (20)	65 % (37)	0.44
Other	7 % ( 7)		4 % ( 2)	9 % ( 5)	0.18
Severe Right Coronaropathy	27 % (28)	103	24 % (11)	30 % (17)	0.13
CI (L/min/m2)	1.5/1.7/2.0	103	1.5/1.6/2.0	1.4/1.7/1.9	-0.14
Preoperative VO2 at peak (ml/min/m2)	9.2/11.0/11.9	78	9.8/11.4/12.4	9.1/10.9/11.7	-0.26
Smoker	46 % (45)	98	41 % (19)	50 % (26)	0.18
Dislipidemia	42 % (43)	103	30 % (14)	51 % (29)	0.43
Hypertension	48 % (49)	103	41 % (19)	53 % (30)	0.23
Preoperative AF	42 % (43)	103	39 % (18)	44 % (25)	0.10
Cancer	10 % (10)	103	4 % ( 2)	14 % ( 8)	0.34
Diabetes	24 % (25)	103	17 % ( 8)	30 % (17)	0.30
Peripheral Vascular disease	24 % (25)	103	22 % (10)	26 % (15)	0.11
COPD	5 % ( 5)	103	4 % ( 2)	5 % ( 3)	0.04
ICD	62 % (64)	103	48 % (22)	74 % (42)	0.55
Reoperation	16 % (16)	103	7 % ( 3)	23 % (13)	0.47
Preoperative platelets (103/mm3)	156/214/285	103	170/237/310	152/205/265	-0.40
BNP	3592/ 6362/12,460	89	2970/ 6173/13,247	3800/ 6961/11,751	-0.06
GFR (mL/min/m3)	50/68/90	96	58/72/90	44/58/90	-0.26
Creatinine (mg/dL)	0.94/1.26/1.58	99	0.93/1.23/1.47	0.96/1.32/1.64	0.30
ASA classification : 3	29 % (30)	103	30 % (14)	28 % (16)	-0.05
4	70 % (72)		67 % (31)	72 % (41)	0.10
5	1 % ( 1)		2 % ( 1)	0 % ( 0)	-0.21
PAPS	37/44/55	94	38/43/55	35/46/56	0.02
EF	16/19/22	103	16/20/23	17/19/21	-0.07
VTD (mL/m2)	109/130/154	100	100/130/156	114/130/154	-0.01
TAPSE	12/14/18	103	12/14/16	13/15/19	0.37
AF right ventricular	25/31/38	103	21/29/38	28/33/38	0.37
IT : 0	2 % ( 2)	103	0 % ( 0)	4 % ( 2)	0.27
1	49 % (50)		48 % (22)	49 % (28)	0.03
2	35 % (36)		35 % (16)	35 % (20)	0.01
3	9 % ( 9)		11 % ( 5)	7 % ( 4)	-0.14
4	6 % ( 6)		7 % ( 3)	5 % ( 3)	-0.05
IM : 0	14 % (14)	103	11 % ( 5)	16 % ( 9)	0.15
1	25 % (26)		15 % ( 7)	33 % (19)	0.43
2	48 % (49)		59 % (27)	39 % (22)	-0.41
3	10 % (10)		11 % ( 5)	9 % ( 5)	-0.07
4	4 % ( 4)		4 % ( 2)	4 % ( 2)	-0.04
IAO : 0	69 % (71)	103	74 % (34)	65 % (37)	-0.20
1	30 % (31)		24 % (11)	35 % (20)	0.25
3	1 % ( 1)		2 % ( 1)	0 % ( 0)	-0.21
Preoperative CVVH	13 % (13)	103	7 % ( 3)	18 % (10)	0.34
More than 1 days with preoperative CVVH	13 % (13)	103	7 % ( 3)	18 % (10)	0.34

covariates that were associated with the outcome and not with the treatment assignment in a small sample size setting is a good choice for the bias-variance trade-off. Patients with missing information were excluded from the initial sample. The final dataset was then composed of 8 variables and 102 subjects. The only subject that was removed was treated with the HeartWare HVAD.

For the present analysis, the Jarvik2000 LVAD group was considered the treatment group. A logistic regression model with only the selected 6 covariates as main effects was used to model the treatment assignment mechanism. The predicted probabilities of being assigned to the treatment group, i.e. the Jarvik2000 group, were used as the individual PS values. Ten different matched sets were formed using PSM, one for each combination of matching with and without replacement and for the different levels of oversampling (from 1 to 5, as in the Monte Carlo simulations). The nearest neighbour with the calliper was used as the matching algorithm, and we set the calliper to 0.2 of the standard deviation of the logit of the PS distribution. The covariate balance of the overall matched sets was investigated using both the ASMD and the OVL. Moreover, we evaluated the goodness of the matching sets by computing the PMT and Proportion of Resampled Controls (PRC). The latter measure was used to check the proportion of controls that were used as candidate matching more than one time when matching was done with replacement. In each matched set, we computed the ATT as the absolute risk reduction of both early and late LDI occurrence attributed to Jarvik2000 LVAD implantation. The 95 % CIs were calculated using both the standard and AI methods, as in the Monte Carlo simulations.

#### Results

**Fig. 3:**
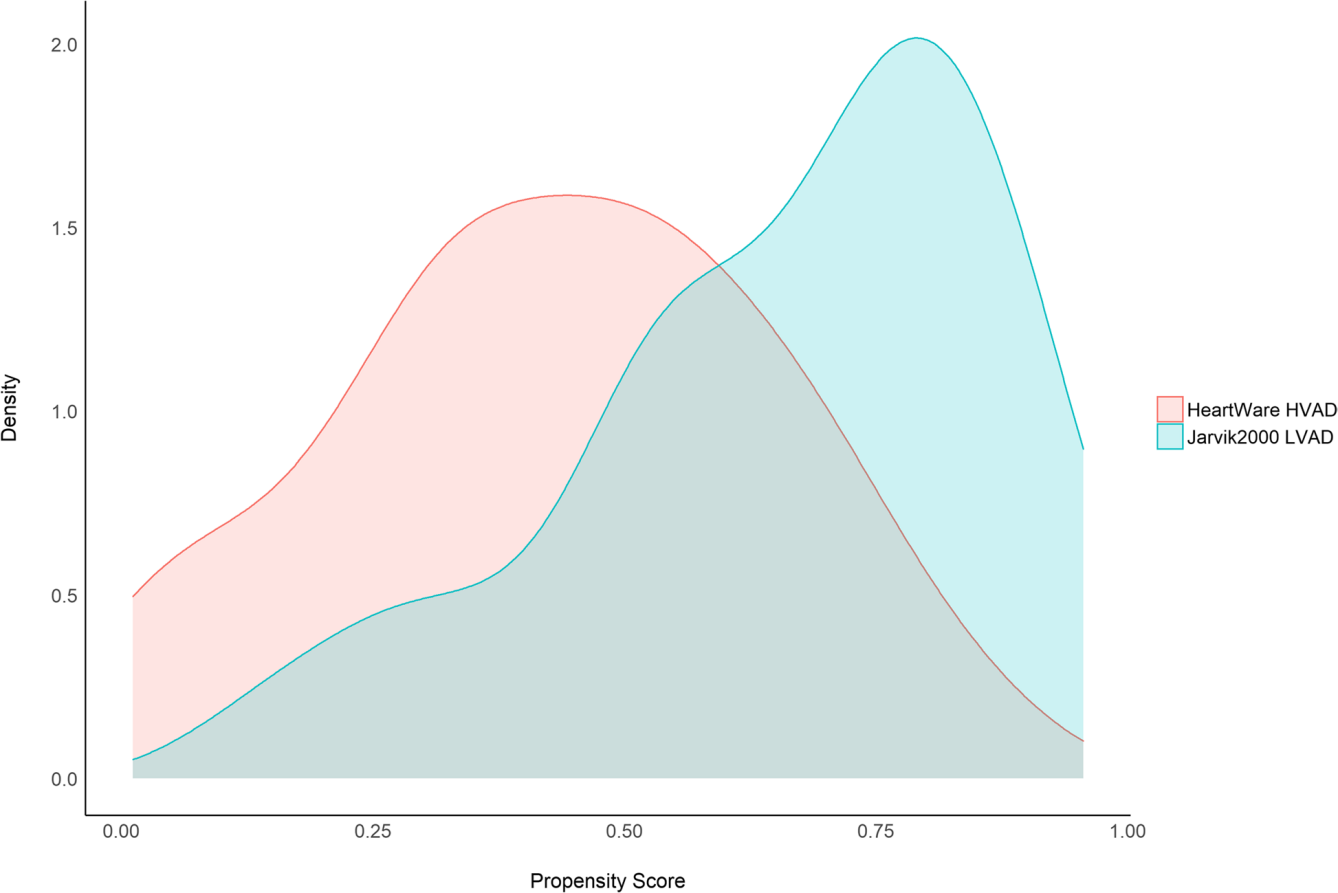
Distributions of estimate Propensity Score (PS) in the unbalanced dataset of the case study. Colors identify the Jarvik2000 LVAD and the HeartWare HVAD groups, which were the treatment and control groups, respectively

The distributions of the estimated PS in the LVAD groups are depicted in Fig. 3. The plot suggests weak-to-moderate common support of baseline characteristics between the two groups. Subjects that underwent Jarvik2000 LVAD implantation had different characteristics on average.

from the patients treated with HeartWare HVAD. As can be seen from Table 1, Jarvik2000 individuals were on average older, had larger body surface area, were treated more frequently with Continuous Veno-Venous Hemofiltration in the preoperative period for more than 1 day, were more frequently assigned to the INTERMACS IV profile, and were more likely to undergo to reintervention.

Table 2 reports the ASMDs, the PMT, and the PRC in each matched set obtained with the different PSM strategies. The OVL results are not shown since they were in line with the ASMDs, as in the Monte Carlo simulations. Classical 1:1 matching without replacement achieved superior performance in terms of balance than 1:1 matching with replacement, with lower ASMD value. However, the more the number of controls matched to each treated unit, the lower the ASMDs values when matching was performed with replacement than when matching was done without replacement. These findings are in line with the Monte Carlo simulations results of the scenario.

**Table 2:** Average Standardized Mean Differences (ASMDs), Proportion of Matched Treated (PMT), and Proportion of Resampled Controls (PRCs) in the matched sets of the case study obtained with all the evaluated PSM strategies

*Replacement*	*Oversampling*	*ASMD*	*PMT*	*PRC*
No	1	0.089	0.526	0.000
	2	0.086	0.316	0.000
	3	0.185	0.193	0.000
	4	0.147	0.140	0.000
	5	0.183	0.105	0.000
Yes	1	0.193	0.895	0.375
	2	0.110	0.737	0.645
	3	0.139	0.596	0.636
	4	0.142	0.579	0.771
	5	0.150	0.509	0.800

with N = 100 and half of the observations assigned to the treatment group, a setting aligned with the LVADs study.

Matching with replacement discarded less treated units from the final set than matching without replacement, as in the Monte Carlo simulations. The matched sets created when matching with replacement are more likely to target the estimand of the reference population of treated. Nevertheless, the proportion of controls that were sampled more than one time increased for higher levels of oversampling. This feature may undermine the reliability of the final treatment effect estimates, as suggested by the higher bias and the lower 95 % NC observed in the Monte Carlo simulations for increasing levels of oversampling when matching was done with replacement. The descriptive statistics of the covariates included in the PS models in the matched samples are provided in the supplementary material.

**Fig. 4:**
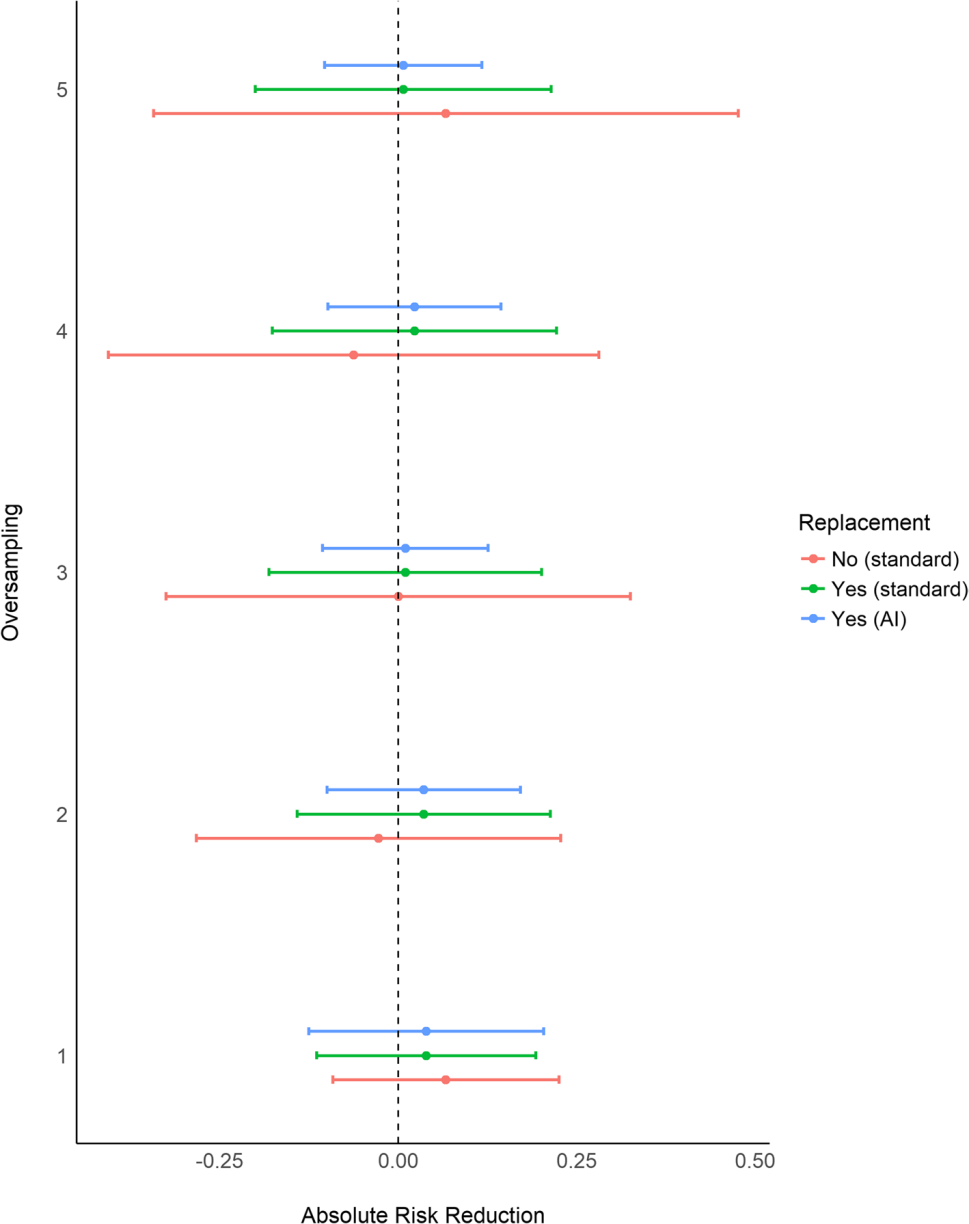
Estimates of the Average Treatment effect on the Treated (ATT), expressed as absolute risk reduction, in the matched sets of the case study obtained with all the evaluated PSM strategies. The dots represent the ATT estimates and the errorbars the 95 % CIs

The ATT estimates are shown in Fig. 4, along with their relative 95 % CIs. No significant differences in terms of both early and late LDI occurrence between the HVAD and JARVIK 2000 were observed, with the 95 % CIs that always included the value of no absolute risk reduction. The length of the CIs obtained with the standard method increased when more controls were matched to each treated, especially when matching was done without replacement. In such settings, the higher number of discarded treated may have increased the variability associated with the final treatment effect estimates. The 95 % CIs computed with the AI method were more similar to each other than the ones obtained with the standard method when matching was done with replacement.

## Discussion

While the performances of matching with oversampling and with replacement have already been studied [20, 32, 42], to the best of our knowledge, the combination of both replacement and oversampling has not been explored so far. Moreover, PSM approaches were usually evaluated using simulation studies or real-word data with large sample sizes. The performances of PSM methods with a low number of enrolled subjects, often encountered in clinical settings, have been assessed by a limited number studies [43, 44]. In the present study, we compared PSM with and without replacement for different levels of oversampling, with a particular focus on small sample size settings, in terms of the overall balance of the matched sets and the performances of the ATT estimator. We employed a popular PSM approach, i.e. NN with a calliper, an algorithm known to perform well in several situations [20]. In addition, we considered the situation with a binary treatment status and a binary outcome, which, to our knowledge, was not assessed in the previous studies that considered PSM in small sample size settings. The approaches were compared using an extensive series of Monte Carlo simulations and a case study from cardiothoracic surgery.

From main simulations, we found that 1:1 matching without replacement achieved a greater overall balance than matching with replacement. Nonetheless, the opposite pattern was observed when the level of oversampling increased, i.e. higher balance for matching with replacement. The overall balance measured with the OVL had the same pattern as the one observed with ASMDs. Using both ASMD and OVL offers several advantages since they provide a measure of balance that simultaneously considers the distributions of all the baseline covariates and not just the separate low-dimensional statistics [34, 45]. Although no information on single covariates are provided, they can be very useful when many PS strategies are implemented and the analyst needs to choose the final set of matched units [31].

As expected, PSM with replacement discarded less treated observations than PSM without replacement. Thus, the use of replacement is more likely to decrease the bias due to incomplete matching. This phenomenon occurs when not all the treated subjects are matched to controls [16]. Incomplete matching narrows the generalizability of the treatment effect estimates only to the subjects that are included in the matched set, and it does not ensure that the new set of individuals is representative of the entire population of treated. Moreover, higher levels of oversampling reduced the PMT, suggesting that it can be tough to find all the pre-specified K matching controls for each treated when K is greater than 1.

Regarding the performances of the ATT estimator, matching with replacement delivers less biased ATT estimates in almost all the settings of the Monte Carlo simulations. These findings are in line with the results of previous studies, which found that matching with replacement increases the number of matched treated which improves generalizability at the expanse of slight bias in not having exact matches [32, 34, 46]. Moreover, the relative bias pattern is consistent with the ASMDs and PMT results: except for 1:1, matching with replacement provided on average more balanced datasets, thus reducing the likelihood of selection bias, and discarded fewer subjects, which may attenuate the bias due to incomplete matching. In contrast to that, oversampling increased the relative bias in all the scenarios except those with low sample size and the number of treated equal or higher than controls. As previously found in the literature, selecting more than one control for each treated subject generally involves a bias-variance tradeoff [34, 46–48]. K greater than 1 usually decreases the variance of the treatment effect estimates at the expense of a higher bias. In the study from Austin (2010), the author recommended matching either 1 or 2 units from the control group [42], which is agreeing with the larger relative bias increase observed in our Monte Carlo simulations when K is equal or greater than 3. Furthermore, the use of oversampling involved a high number of unmatched treated units, increasing the risk of incomplete matching bias. A valuable alternative to oversampling in these situations is represented by the full matching algorithm [49–51]. The algorithm creates a series of matched sets of units containing at least one treated and control by minimizing the distance defined by the estimated PS between treated and control individuals in each matched group. The RMSE results are consistent with the relative bias, suggesting that the ATT estimates obtained when matching is performed with replacement are more precise than those obtained with matching without replacement in most of the scenarios.

The coverage of the 95 % CIs was affected by many factors, such as the settings of the simulations, the PSM strategy, and the methods used to compute them. First of all, when they were calculated using the standard method, matching without replacement resulted in a coverage closer to the nominal value in many scenarios but the ones with large sample size and more treated than control units. NC was lower in almost all scenarios with replacement. The difference was more pronounced when the number of treated was greater than the number of controls. One possible explanation lies in the fact that the standard method does not account for the uncertainty involved in the matching procedure when replacement is used, thus leading to an incorrect NC. The use of the AI method, which in turn accounts for the randomness in the matching process, was evaluated to overcome this issue. As observed in a previous study [52], we found that correct coverage was reached in almost all the scenarios with large sample size. In contrast, in lower sample size settings the coverage worsened for higher levels of oversampling and in situations where more treated than controls are present in the dataset. Increasing levels of oversampling resulted in higher NC in all the settings where the standard method was used. However, higher levels of oversampling are associated with more discarded treated subjects, which may also lead to a reduction of the initial sample size and, thus, to an inflation of the variance associated with treatment effect estimates. This could explain the higher NC values, which in some situations were greater than the 95 % nominal value.

From the second run of simulations, the bias of the ATT estimator was almost negligible in all scenarios, independently of replacement, except when increasing oversampling levels are considered, with better performances for matching with replacement. Matching without replacement resulted in correct coverage only with a 1:1 matching ratio, whereas, for matching with replacement the coverage was good in most of the scenarios when the AI estimator was considered, suggesting the importance of accounting for uncertainty in the matching procedure. Considering both bias and coverage aspects, differences between matching with and without replacement from secondary simulations were less evident than main simulations. The second setup of simulations assumed homogeneity of treatment effect, a simplified situation for which the estimation of ATT might be more straightforward regardless of the strategy used to perform PSM, as particularly evidenced by the relative bias findings. However, in practice, the assumption of treatment effect heterogeneity is often more reasonable and the present findings suggest that matching with replacement can be a reasonable starting point for a broad range of situations.

The overall balance observed in each matched set formed using the case study data was consistent with the results of the Monte Carlo simulations: classical 1:1 matching without replacement returned lower ASMD value than 1:1 matching with replacement. Moreover, higher level of oversampling resulted in lower imbalance in matching with replacement, and higher ASMDs when matching was done without replacement. As in the simulations, PSM with replacement discarded less treated subjects and, both in matching without and with replacement, the PMT was lower for higher K. Furthermore, in matching with replacement, more control units were resampled as candidate matching for higher levels of oversampling. No ATT estimates showed a significant absolute risk reduction of LDI in patients that underwent Jarvik2000 LAVD. When the standard method was used, the width of the 95 % CIs increased when more than one control subject was used as candidate matching. Moreover, 95 % CIs were wider when matching without replacement was performed. The reduction of the initial sample obtained using matching without replacement with increasing K matching controls may have inflated the variance of the ATT estimate, a pattern that likely occurred in the Monte Carlo simulations. In contrast to this, absolute risk reduction estimates obtained with the AI method produced 95 % CIs with lower width than the standard method.

Furthermore, the width was fairly stable across the matched sets of patients. Based on the findings of the Monte Carlo simulations, the most reliable ATT estimates were those obtained with 1:1 or 1:2 matching with replacement and when the AI method was used to compute the 95 % CIs. The estimates suggest that Jarvik2000 LVAD led to an absolute risk reduction of 0.039 (95 % CI -0.125; 0.204) and 0.036 (95 % CI -0.100; 0.171), respectively.

In the case study, patients with missing data were discarded from the analysis. The presence of missing data in baseline covariates is one of the major issues in PS analyses since PS cannot be estimated for those individuals with missing information in baseline characteristics. Classical approaches to deal with missing data in PS analysis are complete case (CC) analysis and missing indicator method (MIND). However, these approaches might be problematic in several situations [53, 54]. Alternatives are represented by methods that include missing values during the estimation of PS [55, 56] and methods based on multiple imputation (MI) [57]. A comparison of several methods for handling missing data in PS analysis with a binary exposure has been recently performed by some studies [58–60]. In the present study, missing data in PS analysis issues were not considered since they are not the focus of the work. We would not expect to observe important differences between methods for handling missing data and PSM strategies given that only one patient was discarded with the CC analysis. However, further research studies are needed to understand how to properly deal with missing data in PSM analysis, especially when sample sizes are small and matching with replacement and oversampling is considered.

Our study has several limitations. First, our Monte Carlo simulations examined a binary outcome, which is similar to the one in the case study. In the future, the proposed approaches should be evaluated in the presence of other types of endpoints, such as time-to-event outcomes, which are of particular interest in clinical practice. Moreover, even if we implemented an extensive set of Monte Carlo simulations, our results should be replicated in different scenarios, such as by considering different proportions of outcome occurrences and different treatment effect magnitudes, which we treated as fixed. Furthermore, other models of the treatment assignment may be explored, such as the use of machine learning techniques [61–63], and the use of different matching algorithms should be considered [50, 64, 65]. Other studies explored the performances of PS methods in small sample size settings [41, 43, 44]. In Pirracchio et al. (2012), the authors found that classical PS-based approaches, such as PSM and using PS as the Inverse Probability of Treatment Weighting (PS-IPTW), led to substantially unbiased treatment effect estimates. However, the difference between settings makes findings hardly comparable. In the future, PSM with replacement and oversampling may be replicated in settings similar to the one proposed by Pirracchio et al. (2012) and compared with more classical PS-based approaches. Furthermore, we compared only two matching ATT estimators: the standard method that accounts for the matched nature of the sample and the AI method. There is considerable debate in the PSM literature about the variance estimation. Some researchers argued that the matching procedure and the uncertainty associated with the PS estimation should not be taken into account and that ATT should be estimated conditional on the covariates, which are assumed to be fixed [66]. Other researchers found that the variance that accounts for the matched nature of the sample provides correct nominal coverage and should be used to compute 95 % CIs [1, 21, 67, 68]. When one wants to account for the uncertainty in the matching procedure, some empirical formulas and bootstrap methods for variance estimation have been proposed [69–72]. Furthermore, there is debate on whether to account for the uncertainty of PS estimation in the variance estimator. Previous studies argued its importance to get valid confidence intervals in the context of PS-IPTW [73, 74]. To the best of our knowledge, the topic has not been explored in-depth so far in the case of PSM, especially when matching approaches alternatives to classical 1:1 without replacement are used [67, 75]. In the present study, we did not consider variance estimators that account for PS estimation uncertainty and we recognize it as a limitation of our work that might explain the incorrect coverage of 95 % CIs obtained in most of the scenarios, especially those with small sample sizes. Future research studies are needed to compare further the performances of variance estimators that account for different sources of uncertainty when PSM is used with replacement and oversampling.

In summary, the treatment effect estimation in small sample size settings remains an open issue. Further work should be aimed in this direction, especially in the medical field, where observational studies are the only alternative when randomization is unethical, and the number of eligible subjects is low. Suppose the researcher wants to estimate the treatment effect using a PSM method. In that case, we recommend using the NN algorithm with replacement and matching no more than 1 or 2 control units to each treated unit. Indeed, the researchers should weight each control unit by the reciprocal of the number of times it was used as a matching candidate. Moreover, the AI method should be used as the variance estimator, since it provided the best NC in most of the settings considered in the Monte Carlo simulations.

## Supplementary Information

**Additional file 1.**

## Data Availability

The dataset of the case study is not publicly available as per research agreement but is available from the corresponding author on reasonable request.
