# Supplementary material for: Oversampling and replacement strategies in propensity score matching: a critical review focused on small sample size in clinical settings
Source: BMC Med Res Methodol. 2021 Nov 22;21:256. doi: 10.1186/s12874-021-01454-z (PMC8609749; doi:10.1186/s12874-021-01454-z)
Supplement: Supplementary file 1 — Additional file 1. [file 12874_2021_1454_MOESM1_ESM.docx]

Table S1. Descriptive statistics of the case study matched sample formed with 1:1 matching without replacement. Continuous variables are represented with median (I and III quartiles) and categorical variables with relative frequencies (percentages). The Standardized Mean Differences (SMDs) on the matched case study sample are reported in the last column of the table.

|  | ***HeartWare HVAD (N=30)*** | ***Jarvik2000 LVAD (N=30)*** | ***Total (N=60)*** | ***SMD*** |
| --- | --- | --- | --- | --- |
| Intermacs IV | 3 (10.0%) | 2 (6.7%) | 5 (8.3%) | -0.03 |
| Age (years) | 58 (52, 64) | 60 (47, 64) | 58 (49, 64) | 0.10 |
| BSA | 2 (2, 2) | 2 (2, 2) | 2 (2, 2) | -0.16 |
| Reoperation | 3 (10.0%) | 5 (16.7%) | 8 (13.3%) | 0.07 |
| EF | 20 (16, 23) | 18 (17, 21) | 19 (17, 22) | -0.03 |
| More than 1 days with preoperative CVVH | 3 (10.0%) | 3 (10.0%) | 6 (10.0%) | 0.00 |

Table S2. Descriptive statistics of the case study matched sample formed with 1:2 matching without replacement. Continuous variables are represented with median (I and III quartiles) and categorical variables with relative frequencies (percentages). The Standardized Mean Differences (SMDs) on the matched case study sample are reported in the last column of the table.

|  | ***HeartWare HVAD (N=18)*** | ***Jarvik2000 LVAD (N=18)*** | ***Total (N=36)*** | ***SMD*** |
| --- | --- | --- | --- | --- |
| Intermacs IV | 1 (5.6%) | 1 (5.6%) | 2 (5.6%) | 0.00 |
| Age (years) | 56 (48, 63) | 57 (45, 62) | 56 (46, 62) | 0.07 |
| BSA | 2 (2, 2) | 2 (2, 2) | 2 (2, 2) | -0.11 |
| Reoperation | 2 (8.3%) | 2 (11.1%) | 4 (9.7%) | 0.03 |
| EF | 20 (15, 22) | 19 (17, 20) | 19 (17, 21) | 0.21 |
| More than 1 days with preoperative CVVH | 2 (8.3%) | 2 (11.1%) | 4 (9.7%) | 0.03 |

Table S3. Descriptive statistics of the case study matched sample formed with 1:3 matching without replacement. Continuous variables are represented with median (I and III quartiles) and categorical variables with relative frequencies (percentages). The Standardized Mean Differences (SMDs) on the matched case study sample are reported in the last column of the table.

|  | ***HeartWare HVAD (N=11)*** | ***Jarvik2000 LVAD (N=11)*** | ***Total (N=22)*** | ***SMD*** |
| --- | --- | --- | --- | --- |
| Intermacs IV | 1 (6.1%) | 0 (0.0%) | 1 (3.0%) | -0.06 |
| Age (years) | 56 (52, 62) | 60 (47, 62) | 57 (50, 62) | 0.09 |
| BSA | 2 (2, 2) | 2 (2, 2) | 2 (2, 2) | 0.09 |
| Reoperation | 0 (3.0%) | 1 (9.1%) | 1 (6.1%) | 0.06 |
| EF | 20 (17, 24) | 20 (18, 20) | 20 (18, 22) | 0.25 |
| More than 1 days with preoperative CVVH | 1 (6.1%) | 2 (18.2%) | 3 (12.1%) | 0.12 |

Table S4. Descriptive statistics of the case study matched sample formed with 1:4 matching without replacement. Continuous variables are represented with median (I and III quartiles) and categorical variables with relative frequencies (percentages). The Standardized Mean Differences (SMDs) on the matched case study sample are reported in the last column of the table.

|  | ***HeartWare HVAD (N=8)*** | ***Jarvik2000 LVAD (N=8)*** | ***Total (N=16)*** | ***SMD*** |
| --- | --- | --- | --- | --- |
| Intermacs IV | 0 (3.1%) | 0 (0.0%) | 0 (1.6%) | -0.03 |
| Age (years) | 56 (50, 64) | 58 (45, 60) | 57 (46, 62) | 0.04 |
| BSA | 2 (2, 2) | 2 (2, 2) | 2 (2, 2) | -0.01 |
| Reoperation | 0 (3.1%) | 0 (0.0%) | 0 (1.6%) | -0.03 |
| EF | 20 (18, 23) | 20 (18, 20) | 20 (18, 22) | 0.08 |
| More than 1 days with preoperative CVVH | 0 (6.2%) | 2 (25.0%) | 2 (15.6%) | 0.19 |

Table S5. Descriptive statistics of the case study matched sample formed with 1:5 matching without replacement. Continuous variables are represented with median (I and III quartiles) and categorical variables with relative frequencies (percentages). The Standardized Mean Differences (SMDs) on the matched case study sample are reported in the last column of the table.

|  | ***HeartWare HVAD (N=6)*** | ***Jarvik2000 LVAD (N=6)*** | ***Total (N=12)*** | ***SMD*** |
| --- | --- | --- | --- | --- |
| Intermacs IV | 1 (10.0%) | 0 (0.0%) | 1 (5.0%) | -0.10 |
| Age (years) | 57 (54, 63) | 58 (48, 62) | 58 (51, 62) | -0.03 |
| BSA | 2 (2, 2) | 2 (2, 2) | 2 (2, 2) | 0.26 |
| Reoperation | 0 (3.3%) | 0 (0.0%) | 0 (1.7%) | -0.03 |
| EF | 20 (18, 23) | 20 (18, 20) | 20 (18, 21) | -0.07 |
| More than 1 days with preoperative CVVH | 0 (3.3%) | 1 (16.7%) | 1 (10.0%) | 0.13 |

Table S6. Descriptive statistics of the case study matched sample formed with 1:1 matching with replacement. Continuous variables are represented with median (I and III quartiles) and categorical variables with relative frequencies (percentages). The Standardized Mean Differences (SMDs) on the matched case study sample are reported in the last column of the table.

|  | ***HeartWare HVAD (N=51)*** | ***Jarvik2000 LVAD (N=51)*** | ***Total (N=102)*** | ***SMD*** |
| --- | --- | --- | --- | --- |
| Intermacs IV | 6 (11.8%) | 9 (17.6%) | 15 (14.7%) | 0.06 |
| Age (years) | 62 (52, 66) | 62 (56, 67) | 62 (55, 66) | 0.22 |
| BSA | 2 (2, 2) | 2 (2, 2) | 2 (2, 2) | -0.09 |
| Reoperation | 16 (31.4%) | 8 (15.7%) | 24 (23.5%) | -0.16 |
| EF | 20 (15, 23) | 19 (17, 21) | 20 (15, 22) | 0.05 |
| More than 1 days with preoperative CVVH | 12 (23.5%) | 8 (15.7%) | 20 (19.6%) | -0.08 |

Table S7. Descriptive statistics of the case study matched sample formed with 1:2 matching with replacement. Continuous variables are represented with median (I and III quartiles) and categorical variables with relative frequencies (percentages). The Standardized Mean Differences (SMDs) on the matched case study sample are reported in the last column of the table.

|  | ***HeartWare HVAD (N=42)*** | ***Jarvik2000 LVAD (N=42)*** | ***Total (N=84)*** | ***SMD*** |
| --- | --- | --- | --- | --- |
| Intermacs IV | 4 (10.7%) | 6 (14.3%) | 10 (12.5%) | 0.04 |
| Age (years) | 58 (52, 64) | 62 (54, 65) | 61 (53, 64) | 0.14 |
| BSA | 2 (2, 2) | 2 (2, 2) | 2 (2, 2) | -0.02 |
| Reoperation | 10 (22.6%) | 5 (11.9%) | 14 (17.3%) | -0.11 |
| EF | 20 (16, 22) | 18 (16, 21) | 19 (16, 21) | 0.02 |
| More than 1 days with preoperative CVVH | 6 (15.5%) | 5 (11.9%) | 12 (13.7%) | -0.04 |

Table S8. Descriptive statistics of the case study matched sample formed with 1:3 matching with replacement. Continuous variables are represented with median (I and III quartiles) and categorical variables with relative frequencies (percentages). The Standardized Mean Differences (SMDs) on the matched case study sample are reported in the last column of the table.

|  | ***HeartWare HVAD (N=34)*** | ***Jarvik2000 LVAD (N=34)*** | ***Total (N=68)*** | ***SMD*** |
| --- | --- | --- | --- | --- |
| Intermacs IV | 6 (18.6%) | 2 (5.9%) | 8 (12.3%) | -0.13 |
| Age (years) | 60 (55, 64) | 61 (54, 65) | 61 (55, 64) | 0.05 |
| BSA | 2 (2, 2) | 2 (2, 2) | 2 (2, 2) | 0.28 |
| Reoperation | 3 (7.8%) | 4 (11.8%) | 7 (9.8%) | 0.04 |
| EF | 20 (18, 23) | 19 (17, 21) | 20 (17, 22) | -0.03 |
| More than 1 days with preoperative CVVH | 3 (9.8%) | 4 (11.8%) | 7 (10.8%) | 0.02 |

Table S9. Descriptive statistics of the case study matched sample formed with 1:4 matching with replacement. Continuous variables are represented with median (I and III quartiles) and categorical variables with relative frequencies (percentages). The Standardized Mean Differences (SMDs) on the matched case study sample are reported in the last column of the table.

|  | ***HeartWare HVAD (N=29)*** | ***Jarvik2000 LVAD (N=29)*** | ***Total (N=58)*** | ***SMD*** |
| --- | --- | --- | --- | --- |
| Intermacs IV | 4 (14.5%) | 1 (3.4%) | 5 (9.0%) | -0.11 |
| Age (years) | 59 (55, 64) | 61 (53, 65) | 60 (55, 65) | -0.04 |
| BSA | 2 (2, 2) | 2 (2, 2) | 2 (2, 2) | 0.30 |
| Reoperation | 2 (5.5%) | 4 (13.8%) | 6 (9.7%) | 0.08 |
| EF | 20 (18, 23) | 19 (17, 21) | 20 (17, 23) | 0.07 |
| More than 1 days with preoperative CVVH | 2 (6.9%) | 2 (6.9%) | 4 (6.9%) | 0.00 |

Table S10. Descriptive statistics of the case study matched sample formed with 1:3 matching with replacement. Continuous variables are represented with median (I and III quartiles) and categorical variables with relative frequencies (percentages). The Standardized Mean Differences (SMDs) on the matched case study sample are reported in the last column of the table.

|  | ***HeartWare HVAD (N=33)*** | ***Jarvik2000 LVAD (N=33)*** | ***Total (N=66)*** | ***SMD*** |
| --- | --- | --- | --- | --- |
| Intermacs IV | 5 (14.4%) | 1 (3.0%) | 6 (8.7%) | -0.11 |
| Age (years) | 59 (55, 65) | 61 (53, 65) | 61 (55, 65) | 0.03 |
| BSA | 2 (2, 2) | 2 (2, 2) | 2 (2, 2) | 0.25 |
| Reoperation | 3 (8.3%) | 4 (12.1%) | 7 (10.2%) | 0.04 |
| EF | 20 (18, 23) | 19 (17, 21) | 20 (17, 22) | 0.05 |
| More than 1 days with preoperative CVVH | 2 (7.6%) | 4 (12.1%) | 6 (9.8%) | 0.05 |

Table S11. Additional parameters that were used in the simulations. For each combination of treatment assignment mechanism and proportion of treated subjects, the table shows the intercept of the treatment assignment model, the intercept of the outcome model, and the coefficient associated with the treatment assignment that was used in the outcome model.

| ***Treatment assignment mechanism*** | ***Proportion of treated subjects*** | ***Intercept of the treatment model*** | ***Intercept of the outcome model*** | ***Treatment’s coefficient of the outcome model*** |
| --- | --- | --- | --- | --- |
| Weak | 0.3 | -1.20 | -1.7 | -1.00 |
| Strong | 0.3 | -0.20 | -1.5 | -1.00 |
| Weak | 0.5 | 0.80 | -1.3 | -1.00 |
| Strong | 0.5 | -1.45 | -1.7 | -0.92 |
| Weak | 0.7 | -0.30 | -1.5 | -0.91 |
| Strong | 0.7 | 0.85 | -1.3 | -0.96 |

Table S12. Relative bias (in %) in each of the 32 simulated scenarios from the primary set of simulations. From left to right the columns report the treatment assignment mechanisms, the proportions of treated subjects, the sample sizes, whether matching was carried out with or without replacement, and the levels of oversampling.

| ***Treatment assignment mechanism*** | ***Proportion of treated*** | ***Sample size*** | ***Replacement*** | ***Oversampling=1*** | ***Oversampling=2*** | ***Oversampling=3*** | ***Oversampling=4*** | ***Oversampling=5*** |
| --- | --- | --- | --- | --- | --- | --- | --- | --- |
| weak | 0.3 | 100 | No | 3.608 | 11.156 | 13.371 | 13.287 | 10.077 |
| weak | 0.3 | 100 | Yes | 7.469 | 1.065 | 5.684 | 8.844 | 10.530 |
| weak | 0.3 | 250 | No | 7.607 | 16.517 | 22.211 | 25.544 | 27.457 |
| weak | 0.3 | 250 | Yes | 0.344 | 3.347 | 5.228 | 6.955 | 8.685 |
| weak | 0.3 | 500 | No | 6.910 | 16.840 | 23.226 | 27.308 | 29.842 |
| weak | 0.3 | 500 | Yes | 0.051 | 1.097 | 2.060 | 3.028 | 4.062 |
| weak | 0.3 | 1,000 | No | 6.725 | 16.684 | 23.490 | 27.534 | 30.194 |
| weak | 0.3 | 1,000 | Yes | 0.601 | 0.853 | 1.327 | 1.735 | 2.110 |
| weak | 0.5 | 100 | No | 13.356 | 18.349 | 16.613 | 12.486 | 7.458 |
| weak | 0.5 | 100 | Yes | 2.487 | 4.878 | 10.031 | 13.524 | 15.834 |
| weak | 0.5 | 250 | No | 18.469 | 26.886 | 29.327 | 30.016 | 29.477 |
| weak | 0.5 | 250 | Yes | 1.567 | 4.463 | 6.824 | 9.004 | 11.248 |
| weak | 0.5 | 500 | No | 18.573 | 28.351 | 31.587 | 33.083 | 33.437 |
| weak | 0.5 | 500 | Yes | 1.009 | 2.240 | 3.463 | 4.653 | 5.875 |
| weak | 0.5 | 1,000 | No | 18.371 | 28.680 | 32.029 | 33.444 | 34.164 |
| weak | 0.5 | 1,000 | Yes | 0.321 | 0.678 | 1.085 | 1.666 | 2.285 |
| weak | 0.7 | 100 | No | 18.823 | 16.978 | 8.894 | 2.803 | 15.915 |
| weak | 0.7 | 100 | Yes | 3.300 | 6.484 | 11.674 | 14.152 | 13.763 |
| weak | 0.7 | 250 | No | 27.492 | 30.036 | 29.349 | 27.232 | 24.138 |
| weak | 0.7 | 250 | Yes | 1.537 | 6.366 | 10.295 | 14.063 | 17.413 |
| weak | 0.7 | 500 | No | 29.189 | 32.730 | 32.973 | 32.844 | 32.267 |
| weak | 0.7 | 500 | Yes | 1.451 | 3.326 | 5.317 | 7.339 | 9.344 |
| weak | 0.7 | 1,000 | No | 29.846 | 33.315 | 34.056 | 33.953 | 34.284 |
| weak | 0.7 | 1,000 | Yes | 0.228 | 1.101 | 2.089 | 3.083 | 3.980 |
| strong | 0.3 | 100 | No | 3.142 | 10.471 | 12.376 | 12.036 | 9.382 |
| strong | 0.3 | 100 | Yes | 11.657 | 2.576 | 3.014 | 7.181 | 10.113 |
| strong | 0.3 | 250 | No | 10.126 | 19.304 | 24.812 | 27.791 | 29.308 |
| strong | 0.3 | 250 | Yes | 2.186 | 1.575 | 4.275 | 6.812 | 8.804 |
| strong | 0.3 | 500 | No | 10.693 | 20.117 | 25.833 | 29.654 | 32.490 |
| strong | 0.3 | 500 | Yes | 0.385 | 0.786 | 1.926 | 3.221 | 4.402 |
| strong | 0.3 | 1,000 | No | 10.695 | 20.184 | 26.128 | 30.025 | 32.875 |
| strong | 0.3 | 1,000 | Yes | 0.384 | 0.004 | 0.548 | 1.075 | 1.629 |
| strong | 0.5 | 100 | No | 14.833 | 20.016 | 19.444 | 14.852 | 9.612 |
| strong | 0.5 | 100 | Yes | 7.360 | 4.557 | 9.811 | 13.893 | 16.276 |
| strong | 0.5 | 250 | No | 22.714 | 30.896 | 33.993 | 34.742 | 34.770 |
| strong | 0.5 | 250 | Yes | 1.143 | 6.174 | 9.439 | 12.415 | 15.137 |
| strong | 0.5 | 500 | No | 23.496 | 32.406 | 36.301 | 38.276 | 39.240 |
| strong | 0.5 | 500 | Yes | 1.283 | 3.406 | 5.567 | 7.307 | 9.058 |
| strong | 0.5 | 1,000 | No | 22.724 | 32.154 | 36.270 | 38.776 | 40.205 |
| strong | 0.5 | 1,000 | Yes | 0.434 | 0.710 | 1.652 | 2.513 | 3.562 |
| strong | 0.7 | 100 | No | 22.779 | 21.129 | 14.076 | 4.514 | 5.436 |
| strong | 0.7 | 100 | Yes | 5.792 | 6.582 | 13.399 | 17.458 | 18.413 |
| strong | 0.7 | 250 | No | 32.433 | 36.090 | 35.635 | 33.935 | 31.467 |
| strong | 0.7 | 250 | Yes | 1.750 | 8.444 | 13.744 | 17.866 | 21.258 |
| strong | 0.7 | 500 | No | 35.273 | 40.497 | 41.545 | 41.341 | 40.865 |
| strong | 0.7 | 500 | Yes | 2.458 | 6.614 | 9.613 | 12.530 | 15.108 |
| strong | 0.7 | 1,000 | No | 35.811 | 41.202 | 42.877 | 43.401 | 43.566 |
| strong | 0.7 | 1,000 | Yes | 0.493 | 2.365 | 4.260 | 6.087 | 7.870 |

Table S13. Root mean squared error in each of the 32 simulated scenarios from the primary set of simulations. From left to right the columns report the treatment assignment mechanisms, the proportions of treated subjects, the sample sizes, whether matching was carried out with or without replacement, and the levels of oversampling.

| ***Treatment assignment mechanism*** | ***Proportion of treated*** | ***Sample size*** | ***Replacement*** | ***Oversampling=1*** | ***Oversampling=2*** | ***Oversampling=3*** | ***Oversampling=4*** | ***Oversampling=5*** |
| --- | --- | --- | --- | --- | --- | --- | --- | --- |
| weak | 0.3 | 100 | No | 0.552 | 1.706 | 2.045 | 2.032 | 1.541 |
| weak | 0.3 | 100 | Yes | 1.142 | 0.163 | 0.869 | 1.353 | 1.610 |
| weak | 0.3 | 250 | No | 1.163 | 2.526 | 3.397 | 3.906 | 4.199 |
| weak | 0.3 | 250 | Yes | 0.053 | 0.512 | 0.800 | 1.064 | 1.328 |
| weak | 0.3 | 500 | No | 1.057 | 2.575 | 3.552 | 4.176 | 4.564 |
| weak | 0.3 | 500 | Yes | 0.008 | 0.168 | 0.315 | 0.463 | 0.621 |
| weak | 0.3 | 1,000 | No | 1.028 | 2.551 | 3.592 | 4.211 | 4.617 |
| weak | 0.3 | 1,000 | Yes | 0.092 | 0.130 | 0.203 | 0.265 | 0.323 |
| weak | 0.5 | 100 | No | 2.035 | 2.795 | 2.531 | 1.902 | 1.136 |
| weak | 0.5 | 100 | Yes | 0.379 | 0.743 | 1.528 | 2.060 | 2.412 |
| weak | 0.5 | 250 | No | 2.814 | 4.096 | 4.468 | 4.573 | 4.491 |
| weak | 0.5 | 250 | Yes | 0.239 | 0.680 | 1.040 | 1.372 | 1.714 |
| weak | 0.5 | 500 | No | 2.830 | 4.319 | 4.812 | 5.040 | 5.094 |
| weak | 0.5 | 500 | Yes | 0.154 | 0.341 | 0.528 | 0.709 | 0.895 |
| weak | 0.5 | 1,000 | No | 2.799 | 4.370 | 4.880 | 5.095 | 5.205 |
| weak | 0.5 | 1,000 | Yes | 0.049 | 0.103 | 0.165 | 0.254 | 0.348 |
| weak | 0.7 | 100 | No | 2.892 | 2.608 | 1.366 | 0.431 | 2.439 |
| weak | 0.7 | 100 | Yes | 0.507 | 0.996 | 1.793 | 2.174 | 2.110 |
| weak | 0.7 | 250 | No | 4.223 | 4.614 | 4.509 | 4.183 | 3.708 |
| weak | 0.7 | 250 | Yes | 0.236 | 0.978 | 1.582 | 2.160 | 2.675 |
| weak | 0.7 | 500 | No | 4.484 | 5.028 | 5.065 | 5.046 | 4.957 |
| weak | 0.7 | 500 | Yes | 0.223 | 0.511 | 0.817 | 1.127 | 1.435 |
| weak | 0.7 | 1,000 | No | 4.585 | 5.118 | 5.232 | 5.216 | 5.267 |
| weak | 0.7 | 1,000 | Yes | 0.035 | 0.169 | 0.321 | 0.474 | 0.611 |
| strong | 0.3 | 100 | No | 0.491 | 1.636 | 1.933 | 1.880 | 1.466 |
| strong | 0.3 | 100 | Yes | 1.821 | 0.402 | 0.471 | 1.122 | 1.580 |
| strong | 0.3 | 250 | No | 1.582 | 3.016 | 3.876 | 4.341 | 4.578 |
| strong | 0.3 | 250 | Yes | 0.341 | 0.246 | 0.668 | 1.064 | 1.375 |
| strong | 0.3 | 500 | No | 1.670 | 3.143 | 4.036 | 4.632 | 5.076 |
| strong | 0.3 | 500 | Yes | 0.060 | 0.123 | 0.301 | 0.503 | 0.688 |
| strong | 0.3 | 1,000 | No | 1.671 | 3.153 | 4.082 | 4.690 | 5.136 |
| strong | 0.3 | 1,000 | Yes | 0.060 | 0.001 | 0.086 | 0.168 | 0.254 |
| strong | 0.5 | 100 | No | 2.224 | 3.002 | 2.916 | 2.227 | 1.441 |
| strong | 0.5 | 100 | Yes | 1.104 | 0.683 | 1.471 | 2.084 | 2.441 |
| strong | 0.5 | 250 | No | 3.406 | 4.633 | 5.098 | 5.210 | 5.214 |
| strong | 0.5 | 250 | Yes | 0.171 | 0.926 | 1.415 | 1.862 | 2.270 |
| strong | 0.5 | 500 | No | 3.524 | 4.860 | 5.444 | 5.740 | 5.885 |
| strong | 0.5 | 500 | Yes | 0.192 | 0.511 | 0.835 | 1.096 | 1.358 |
| strong | 0.5 | 1,000 | No | 3.408 | 4.822 | 5.439 | 5.815 | 6.029 |
| strong | 0.5 | 1,000 | Yes | 0.065 | 0.106 | 0.248 | 0.377 | 0.534 |
| strong | 0.7 | 100 | No | 3.464 | 3.213 | 2.140 | 0.686 | 0.824 |
| strong | 0.7 | 100 | Yes | 0.881 | 1.001 | 2.037 | 2.654 | 2.791 |
| strong | 0.7 | 250 | No | 4.931 | 5.488 | 5.418 | 5.160 | 4.785 |
| strong | 0.7 | 250 | Yes | 0.266 | 1.284 | 2.090 | 2.717 | 3.232 |
| strong | 0.7 | 500 | No | 5.363 | 6.158 | 6.317 | 6.286 | 6.214 |
| strong | 0.7 | 500 | Yes | 0.374 | 1.006 | 1.462 | 1.905 | 2.297 |
| strong | 0.7 | 1,000 | No | 5.445 | 6.265 | 6.519 | 6.599 | 6.624 |
| strong | 0.7 | 1,000 | Yes | 0.075 | 0.360 | 0.648 | 0.925 | 1.197 |

Table S14. Nominal coverage of the 95% CIs in simulations obtained using the standard error estimator that accounts for the matched nature of the sample from the primary set of simulations. From left to right the columns report the treatment assignment mechanisms, the proportions of treated subjects, the sample sizes, whether matching was carried out with or without replacement, and the levels of oversampling.

| ***Treatment assignment mechanism*** | ***Proportion of treated*** | ***Sample size*** | ***Replacement*** | ***Oversampling=1*** | ***Oversampling=2*** | ***Oversampling=3*** | ***Oversampling=4*** | ***Oversampling=5*** |
| --- | --- | --- | --- | --- | --- | --- | --- | --- |
| weak | 0.3 | 100 | No | 0.974 | 0.995 | 0.998 | 0.999 | 0.999 |
| weak | 0.3 | 100 | Yes | 0.927 | 0.968 | 0.982 | 0.989 | 0.991 |
| weak | 0.3 | 250 | No | 0.948 | 0.983 | 0.994 | 0.998 | 1.000 |
| weak | 0.3 | 250 | Yes | 0.870 | 0.922 | 0.943 | 0.954 | 0.960 |
| weak | 0.3 | 500 | No | 0.941 | 0.959 | 0.958 | 0.960 | 0.967 |
| weak | 0.3 | 500 | Yes | 0.857 | 0.908 | 0.926 | 0.939 | 0.947 |
| weak | 0.3 | 1,000 | No | 0.940 | 0.931 | 0.911 | 0.902 | 0.905 |
| weak | 0.3 | 1,000 | Yes | 0.865 | 0.912 | 0.927 | 0.934 | 0.940 |
| weak | 0.5 | 100 | No | 0.975 | 0.996 | 0.998 | 0.997 | 0.996 |
| weak | 0.5 | 100 | Yes | 0.870 | 0.927 | 0.952 | 0.967 | 0.976 |
| weak | 0.5 | 250 | No | 0.916 | 0.955 | 0.991 | 0.999 | 1.000 |
| weak | 0.5 | 250 | Yes | 0.768 | 0.833 | 0.860 | 0.876 | 0.884 |
| weak | 0.5 | 500 | No | 0.887 | 0.895 | 0.923 | 0.953 | 0.977 |
| weak | 0.5 | 500 | Yes | 0.756 | 0.818 | 0.842 | 0.861 | 0.868 |
| weak | 0.5 | 1,000 | No | 0.831 | 0.780 | 0.814 | 0.864 | 0.903 |
| weak | 0.5 | 1,000 | Yes | 0.767 | 0.819 | 0.846 | 0.857 | 0.864 |
| weak | 0.7 | 100 | No | 0.969 | 0.991 | 0.985 | 0.969 | 0.933 |
| weak | 0.7 | 100 | Yes | 0.778 | 0.864 | 0.906 | 0.931 | 0.931 |
| weak | 0.7 | 250 | No | 0.884 | 0.979 | 0.997 | 0.999 | 0.999 |
| weak | 0.7 | 250 | Yes | 0.634 | 0.714 | 0.742 | 0.760 | 0.770 |
| weak | 0.7 | 500 | No | 0.799 | 0.898 | 0.958 | 0.991 | 0.999 |
| weak | 0.7 | 500 | Yes | 0.617 | 0.687 | 0.713 | 0.732 | 0.741 |
| weak | 0.7 | 1,000 | No | 0.644 | 0.777 | 0.875 | 0.924 | 0.953 |
| weak | 0.7 | 1,000 | Yes | 0.617 | 0.670 | 0.695 | 0.713 | 0.726 |
| strong | 0.3 | 100 | No | 0.974 | 0.996 | 0.998 | 0.999 | 0.999 |
| strong | 0.3 | 100 | Yes | 0.915 | 0.961 | 0.977 | 0.986 | 0.991 |
| strong | 0.3 | 250 | No | 0.960 | 0.990 | 0.996 | 0.999 | 1.000 |
| strong | 0.3 | 250 | Yes | 0.859 | 0.910 | 0.933 | 0.950 | 0.960 |
| strong | 0.3 | 500 | No | 0.938 | 0.953 | 0.960 | 0.965 | 0.976 |
| strong | 0.3 | 500 | Yes | 0.817 | 0.875 | 0.903 | 0.917 | 0.925 |
| strong | 0.3 | 1,000 | No | 0.921 | 0.922 | 0.909 | 0.904 | 0.904 |
| strong | 0.3 | 1,000 | Yes | 0.818 | 0.868 | 0.890 | 0.901 | 0.912 |
| strong | 0.5 | 100 | No | 0.974 | 0.993 | 0.996 | 0.997 | 0.995 |
| strong | 0.5 | 100 | Yes | 0.866 | 0.925 | 0.954 | 0.964 | 0.974 |
| strong | 0.5 | 250 | No | 0.914 | 0.968 | 0.993 | 0.998 | 0.999 |
| strong | 0.5 | 250 | Yes | 0.726 | 0.803 | 0.835 | 0.852 | 0.864 |
| strong | 0.5 | 500 | No | 0.864 | 0.880 | 0.910 | 0.944 | 0.976 |
| strong | 0.5 | 500 | Yes | 0.700 | 0.772 | 0.800 | 0.819 | 0.830 |
| strong | 0.5 | 1,000 | No | 0.800 | 0.765 | 0.790 | 0.824 | 0.855 |
| strong | 0.5 | 1,000 | Yes | 0.692 | 0.750 | 0.783 | 0.797 | 0.813 |
| strong | 0.7 | 100 | No | 0.960 | 0.984 | 0.974 | 0.950 | 0.905 |
| strong | 0.7 | 100 | Yes | 0.774 | 0.855 | 0.896 | 0.919 | 0.916 |
| strong | 0.7 | 250 | No | 0.868 | 0.966 | 0.992 | 0.997 | 0.997 |
| strong | 0.7 | 250 | Yes | 0.589 | 0.669 | 0.705 | 0.726 | 0.736 |
| strong | 0.7 | 500 | No | 0.748 | 0.836 | 0.926 | 0.978 | 0.995 |
| strong | 0.7 | 500 | Yes | 0.545 | 0.625 | 0.658 | 0.673 | 0.689 |
| strong | 0.7 | 1,000 | No | 0.559 | 0.651 | 0.763 | 0.842 | 0.898 |
| strong | 0.7 | 1,000 | Yes | 0.520 | 0.583 | 0.615 | 0.639 | 0.648 |

Table S15. Nominal coverage of the 95% CIs in simulations obtained using the Abadie-Imbens standard error from the primary set of simulations. From left to right the columns report the treatment assignment mechanisms, the proportions of treated subjects, the sample sizes, whether matching was carried out with or without replacement, and the levels of oversampling.

| ***Treatment assignment mechanism*** | ***Proportion of treated*** | ***Sample size*** | ***Oversampling=1*** | ***Oversampling=2*** | ***Oversampling=3*** | ***Oversampling=4*** | ***Oversampling=5*** |
| --- | --- | --- | --- | --- | --- | --- | --- |
| weak | 0.3 | 100 | 0.948 | 0.932 | 0.903 | 0.864 | 0.824 |
| weak | 0.3 | 250 | 0.946 | 0.927 | 0.915 | 0.900 | 0.887 |
| weak | 0.3 | 500 | 0.933 | 0.933 | 0.928 | 0.926 | 0.922 |
| weak | 0.3 | 1,000 | 0.939 | 0.941 | 0.942 | 0.938 | 0.936 |
| weak | 0.5 | 100 | 0.921 | 0.880 | 0.819 | 0.751 | 0.667 |
| weak | 0.5 | 250 | 0.926 | 0.902 | 0.886 | 0.866 | 0.845 |
| weak | 0.5 | 500 | 0.930 | 0.924 | 0.917 | 0.912 | 0.902 |
| weak | 0.5 | 1,000 | 0.940 | 0.937 | 0.937 | 0.934 | 0.930 |
| weak | 0.7 | 100 | 0.861 | 0.788 | 0.675 | 0.555 | 0.448 |
| weak | 0.7 | 250 | 0.894 | 0.866 | 0.828 | 0.784 | 0.735 |
| weak | 0.7 | 500 | 0.908 | 0.902 | 0.892 | 0.876 | 0.853 |
| weak | 0.7 | 1,000 | 0.927 | 0.925 | 0.924 | 0.920 | 0.915 |
| strong | 0.3 | 100 | 0.927 | 0.908 | 0.877 | 0.829 | 0.775 |
| strong | 0.3 | 250 | 0.940 | 0.916 | 0.895 | 0.875 | 0.856 |
| strong | 0.3 | 500 | 0.924 | 0.918 | 0.917 | 0.910 | 0.899 |
| strong | 0.3 | 1,000 | 0.933 | 0.930 | 0.928 | 0.926 | 0.922 |
| strong | 0.5 | 100 | 0.895 | 0.847 | 0.775 | 0.690 | 0.621 |
| strong | 0.5 | 250 | 0.911 | 0.883 | 0.851 | 0.817 | 0.784 |
| strong | 0.5 | 500 | 0.907 | 0.908 | 0.895 | 0.881 | 0.866 |
| strong | 0.5 | 1,000 | 0.926 | 0.927 | 0.923 | 0.917 | 0.908 |
| strong | 0.7 | 100 | 0.808 | 0.733 | 0.620 | 0.520 | 0.428 |
| strong | 0.7 | 250 | 0.860 | 0.815 | 0.763 | 0.709 | 0.651 |
| strong | 0.7 | 500 | 0.878 | 0.867 | 0.844 | 0.815 | 0.785 |
| strong | 0.7 | 1,000 | 0.897 | 0.900 | 0.890 | 0.881 | 0.866 |

Table S16. Parameters of the models used in the secondary simulations. For each combination of treatment assignment mechanism and proportion of treated subjects, the table shows the intercept of the treatment assignment model, the intercept of the outcome model, the coefficient associated with the treatment assignment that was used in the outcome model, and the coefficients of the outcome model associated with the confounders.

| ***Treatment assignment mechanism*** | ***Proportion of treated subjects*** | ***Intercept of the treatment model*** | ***Intercept of the outcome model*** | ***Treatment’s coefficient of the outcome model*** | ***Covariates coefficients of the outcome model*** |
| --- | --- | --- | --- | --- | --- |
| Weak | 0.3 | -1.20 | 0.18 | -0.15 | (0.25, -0.01, 0.02, 0.003, -0.005, -0.001) |
| Strong | 0.3 | -0.20 | 0.22 | -0.15 | 0.25, -0.01, 0.02, 0.003, -0.005, -0.001) |
| Weak | 0.5 | 0.80 | 0.24 | -0.15 | 0.25, -0.01, 0.02, 0.003, -0.005, -0.001) |
| Strong | 0.5 | -1.45 | 0.19 | -0.15 | 0.25, -0.01, 0.02, 0.003, -0.005, -0.001) |
| Weak | 0.7 | -0.30 | 0.21 | -0.15 | 0.25, -0.01, 0.02, 0.003, -0.005, -0.001) |
| Strong | 0.7 | 0.85 | 0.24 | -0.15 | 0.25, -0.01, 0.02, 0.003, -0.005, -0.001) |

Table S17. Relative bias (in %) in each of the 32 simulated scenarios from the secondary set of simulations. From left to right the columns report the treatment assignment mechanisms, the proportions of treated subjects, the sample sizes, whether matching was carried out with or without replacement, and the levels of oversampling.

| ***Treatment assignment mechanism*** | ***Proportion of treated*** | ***Sample size*** | ***Replacement*** | ***Oversampling=1*** | ***Oversampling=2*** | ***Oversampling=3*** | ***Oversampling=4*** | ***Oversampling=5*** |
| --- | --- | --- | --- | --- | --- | --- | --- | --- |
| weak | 0.3 | 100 | No | 5.521 | 4.705 | 6.593 | 8.305 | 11.571 |
| weak | 0.3 | 100 | Yes | 9.002 | 5.435 | 4.536 | 4.959 | 5.531 |
| weak | 0.3 | 250 | No | 0.117 | 0.206 | 0.321 | 0.639 | 0.764 |
| weak | 0.3 | 250 | Yes | 0.901 | 0.110 | 0.029 | 0.117 | 0.203 |
| weak | 0.3 | 500 | No | 0.129 | 0.359 | 0.483 | 0.383 | 0.207 |
| weak | 0.3 | 500 | Yes | 0.108 | 0.006 | 0.324 | 0.349 | 0.463 |
| weak | 0.3 | 1,000 | No | 0.029 | 0.131 | 0.259 | 0.175 | 0.137 |
| weak | 0.3 | 1,000 | Yes | 0.283 | 0.389 | 0.440 | 0.302 | 0.202 |
| weak | 0.5 | 100 | No | 4.760 | 5.975 | 9.063 | 15.072 | 20.594 |
| weak | 0.5 | 100 | Yes | 9.595 | 6.420 | 6.436 | 6.428 | 6.884 |
| weak | 0.5 | 250 | No | 0.404 | 0.360 | 0.656 | 0.937 | 1.985 |
| weak | 0.5 | 250 | Yes | 1.690 | 1.239 | 0.906 | 0.471 | 0.486 |
| weak | 0.5 | 500 | No | 0.180 | 0.147 | 0.142 | 0.100 | 0.151 |
| weak | 0.5 | 500 | Yes | 0.670 | 0.741 | 0.624 | 0.611 | 0.553 |
| weak | 0.5 | 1,000 | No | 0.322 | 0.176 | 0.274 | 0.322 | 0.354 |
| weak | 0.5 | 1,000 | Yes | 0.572 | 0.732 | 0.668 | 0.661 | 0.643 |
| weak | 0.7 | 100 | No | 6.717 | 12.371 | 23.812 | 36.849 | 55.960 |
| weak | 0.7 | 100 | Yes | 11.438 | 7.191 | 7.183 | 9.369 | 14.436 |
| weak | 0.7 | 250 | No | 0.001 | 1.366 | 2.958 | 5.735 | 8.801 |
| weak | 0.7 | 250 | Yes | 1.454 | 0.870 | 0.240 | 0.147 | 0.224 |
| weak | 0.7 | 500 | No | 0.410 | 0.034 | 0.493 | 0.850 | 1.609 |
| weak | 0.7 | 500 | Yes | 0.674 | 0.903 | 0.804 | 0.770 | 0.854 |
| weak | 0.7 | 1,000 | No | 0.083 | 0.183 | 0.233 | 0.371 | 0.299 |
| weak | 0.7 | 1,000 | Yes | 0.457 | 0.380 | 0.441 | 0.543 | 0.501 |
| strong | 0.3 | 100 | No | 6.014 | 5.918 | 7.375 | 10.223 | 13.102 |
| strong | 0.3 | 100 | Yes | 12.134 | 7.067 | 6.245 | 6.086 | 6.025 |
| strong | 0.3 | 250 | No | 0.212 | 0.150 | 0.114 | 0.011 | 0.134 |
| strong | 0.3 | 250 | Yes | 2.096 | 0.512 | 0.027 | 0.148 | 0.143 |
| strong | 0.3 | 500 | No | 1.188 | 1.361 | 1.507 | 1.453 | 1.141 |
| strong | 0.3 | 500 | Yes | 0.861 | 0.953 | 0.939 | 0.877 | 1.055 |
| strong | 0.3 | 1,000 | No | 0.816 | 0.799 | 0.743 | 0.676 | 0.613 |
| strong | 0.3 | 1,000 | Yes | 0.095 | 0.111 | 0.003 | 0.124 | 0.260 |
| strong | 0.5 | 100 | No | 3.726 | 5.299 | 9.078 | 13.849 | 20.414 |
| strong | 0.5 | 100 | Yes | 9.168 | 5.221 | 4.019 | 4.735 | 5.407 |
| strong | 0.5 | 250 | No | 0.589 | 0.216 | 0.682 | 1.633 | 2.655 |
| strong | 0.5 | 250 | Yes | 0.693 | 0.068 | 0.183 | 0.224 | 0.596 |
| strong | 0.5 | 500 | No | 1.043 | 0.806 | 0.615 | 0.594 | 0.519 |
| strong | 0.5 | 500 | Yes | 0.172 | 0.568 | 0.602 | 0.397 | 0.410 |
| strong | 0.5 | 1,000 | No | 0.764 | 0.631 | 0.540 | 0.572 | 0.446 |
| strong | 0.5 | 1,000 | Yes | 0.012 | 0.151 | 0.100 | 0.088 | 0.044 |
| strong | 0.7 | 100 | No | 7.335 | 12.828 | 21.622 | 34.808 | 51.471 |
| strong | 0.7 | 100 | Yes | 15.508 | 9.503 | 9.313 | 10.990 | 14.366 |
| strong | 0.7 | 250 | No | 0.808 | 2.355 | 4.035 | 6.321 | 8.599 |
| strong | 0.7 | 250 | Yes | 4.229 | 2.677 | 2.279 | 2.291 | 1.858 |
| strong | 0.7 | 500 | No | 0.162 | 0.063 | 0.353 | 0.407 | 0.753 |
| strong | 0.7 | 500 | Yes | 1.807 | 2.039 | 1.726 | 1.490 | 1.348 |
| strong | 0.7 | 1,000 | No | 0.271 | 0.151 | 0.110 | 0.429 | 0.506 |
| strong | 0.7 | 1,000 | Yes | 0.954 | 1.037 | 0.804 | 0.865 | 0.936 |

Table S18. Root mean squared error in each of the 32 simulated scenarios from the secondary set of simulations. From left to right the columns report the treatment assignment mechanisms, the proportions of treated subjects, the sample sizes, whether matching was carried out with or without replacement, and the levels of oversampling.

| ***Treatment assignment mechanism*** | ***Proportion of treated*** | ***Sample size*** | ***Replacement*** | ***Oversampling=1*** | ***Oversampling=2*** | ***Oversampling=3*** | ***Oversampling=4*** | ***Oversampling=5*** |
| --- | --- | --- | --- | --- | --- | --- | --- | --- |
| weak | 0.3 | 100 | No | 0.828 | 0.706 | 0.989 | 1.246 | 1.736 |
| weak | 0.3 | 100 | Yes | 1.350 | 0.815 | 0.680 | 0.744 | 0.830 |
| weak | 0.3 | 250 | No | 0.018 | 0.031 | 0.048 | 0.096 | 0.115 |
| weak | 0.3 | 250 | Yes | 0.135 | 0.016 | 0.004 | 0.018 | 0.030 |
| weak | 0.3 | 500 | No | 0.019 | 0.054 | 0.072 | 0.057 | 0.031 |
| weak | 0.3 | 500 | Yes | 0.016 | 0.001 | 0.049 | 0.052 | 0.069 |
| weak | 0.3 | 1,000 | No | 0.004 | 0.020 | 0.039 | 0.026 | 0.021 |
| weak | 0.3 | 1,000 | Yes | 0.042 | 0.058 | 0.066 | 0.045 | 0.030 |
| weak | 0.5 | 100 | No | 0.714 | 0.896 | 1.359 | 2.261 | 3.089 |
| weak | 0.5 | 100 | Yes | 1.439 | 0.963 | 0.965 | 0.964 | 1.033 |
| weak | 0.5 | 250 | No | 0.061 | 0.054 | 0.098 | 0.141 | 0.298 |
| weak | 0.5 | 250 | Yes | 0.254 | 0.186 | 0.136 | 0.071 | 0.073 |
| weak | 0.5 | 500 | No | 0.027 | 0.022 | 0.021 | 0.015 | 0.023 |
| weak | 0.5 | 500 | Yes | 0.100 | 0.111 | 0.094 | 0.092 | 0.083 |
| weak | 0.5 | 1,000 | No | 0.048 | 0.026 | 0.041 | 0.048 | 0.053 |
| weak | 0.5 | 1,000 | Yes | 0.086 | 0.110 | 0.100 | 0.099 | 0.096 |
| weak | 0.7 | 100 | No | 1.008 | 1.856 | 3.572 | 5.527 | 8.369 |
| weak | 0.7 | 100 | Yes | 1.716 | 1.079 | 1.077 | 1.405 | 2.159 |
| weak | 0.7 | 250 | No | 0.000 | 0.205 | 0.444 | 0.860 | 1.320 |
| weak | 0.7 | 250 | Yes | 0.218 | 0.131 | 0.036 | 0.022 | 0.034 |
| weak | 0.7 | 500 | No | 0.062 | 0.005 | 0.074 | 0.128 | 0.241 |
| weak | 0.7 | 500 | Yes | 0.101 | 0.135 | 0.121 | 0.115 | 0.128 |
| weak | 0.7 | 1,000 | No | 0.012 | 0.027 | 0.035 | 0.056 | 0.045 |
| weak | 0.7 | 1,000 | Yes | 0.069 | 0.057 | 0.066 | 0.081 | 0.075 |
| strong | 0.3 | 100 | No | 0.902 | 0.888 | 1.106 | 1.533 | 1.965 |
| strong | 0.3 | 100 | Yes | 1.820 | 1.060 | 0.937 | 0.913 | 0.904 |
| strong | 0.3 | 250 | No | 0.032 | 0.023 | 0.017 | 0.002 | 0.020 |
| strong | 0.3 | 250 | Yes | 0.314 | 0.077 | 0.004 | 0.022 | 0.021 |
| strong | 0.3 | 500 | No | 0.178 | 0.204 | 0.226 | 0.218 | 0.171 |
| strong | 0.3 | 500 | Yes | 0.129 | 0.143 | 0.141 | 0.132 | 0.158 |
| strong | 0.3 | 1,000 | No | 0.122 | 0.120 | 0.111 | 0.101 | 0.092 |
| strong | 0.3 | 1,000 | Yes | 0.014 | 0.017 | 0.000 | 0.019 | 0.039 |
| strong | 0.5 | 100 | No | 0.559 | 0.795 | 1.362 | 2.077 | 3.062 |
| strong | 0.5 | 100 | Yes | 1.375 | 0.783 | 0.603 | 0.710 | 0.811 |
| strong | 0.5 | 250 | No | 0.088 | 0.032 | 0.102 | 0.245 | 0.398 |
| strong | 0.5 | 250 | Yes | 0.104 | 0.010 | 0.027 | 0.034 | 0.089 |
| strong | 0.5 | 500 | No | 0.156 | 0.121 | 0.092 | 0.089 | 0.078 |
| strong | 0.5 | 500 | Yes | 0.026 | 0.085 | 0.090 | 0.060 | 0.061 |
| strong | 0.5 | 1,000 | No | 0.115 | 0.095 | 0.081 | 0.086 | 0.067 |
| strong | 0.5 | 1,000 | Yes | 0.002 | 0.023 | 0.015 | 0.013 | 0.007 |
| strong | 0.7 | 100 | No | 1.100 | 1.924 | 3.243 | 5.221 | 7.700 |
| strong | 0.7 | 100 | Yes | 2.326 | 1.426 | 1.397 | 1.648 | 2.149 |
| strong | 0.7 | 250 | No | 0.121 | 0.353 | 0.605 | 0.948 | 1.290 |
| strong | 0.7 | 250 | Yes | 0.634 | 0.402 | 0.342 | 0.344 | 0.279 |
| strong | 0.7 | 500 | No | 0.024 | 0.009 | 0.053 | 0.061 | 0.113 |
| strong | 0.7 | 500 | Yes | 0.271 | 0.306 | 0.259 | 0.224 | 0.202 |
| strong | 0.7 | 1,000 | No | 0.041 | 0.023 | 0.016 | 0.064 | 0.076 |
| strong | 0.7 | 1,000 | Yes | 0.143 | 0.156 | 0.121 | 0.130 | 0.140 |

Table S19. Nominal coverage of the 95% CIs in simulations obtained using the standard error estimator that accounts for the matched nature of the sample from the secondary set of simulations. From left to right the columns report the treatment assignment mechanisms, the proportions of treated subjects, the sample sizes, whether matching was carried out with or without replacement, and the levels of oversampling.

| ***Treatment assignment mechanism*** | ***Proportion of treated*** | ***Sample size*** | ***Replacement*** | ***Oversampling=1*** | ***Oversampling=2*** | ***Oversampling=3*** | ***Oversampling=4*** | ***Oversampling=5*** |
| --- | --- | --- | --- | --- | --- | --- | --- | --- |
| weak | 0.3 | 100 | No | 0.964 | 0.991 | 0.998 | 1.000 | 1.000 |
| weak | 0.3 | 100 | Yes | 0.912 | 0.959 | 0.977 | 0.984 | 0.990 |
| weak | 0.3 | 250 | No | 0.955 | 0.989 | 0.997 | 0.999 | 0.999 |
| weak | 0.3 | 250 | Yes | 0.862 | 0.924 | 0.945 | 0.960 | 0.967 |
| weak | 0.3 | 500 | No | 0.950 | 0.985 | 0.992 | 0.996 | 0.997 |
| weak | 0.3 | 500 | Yes | 0.858 | 0.915 | 0.939 | 0.949 | 0.958 |
| weak | 0.3 | 1,000 | No | 0.956 | 0.988 | 0.995 | 0.997 | 0.998 |
| weak | 0.3 | 1,000 | Yes | 0.871 | 0.919 | 0.937 | 0.949 | 0.954 |
| weak | 0.5 | 100 | No | 0.968 | 0.989 | 0.997 | 0.999 | 0.999 |
| weak | 0.5 | 100 | Yes | 0.863 | 0.925 | 0.950 | 0.962 | 0.971 |
| weak | 0.5 | 250 | No | 0.952 | 0.989 | 0.995 | 0.997 | 0.998 |
| weak | 0.5 | 250 | Yes | 0.782 | 0.852 | 0.885 | 0.904 | 0.915 |
| weak | 0.5 | 500 | No | 0.958 | 0.984 | 0.992 | 0.996 | 0.999 |
| weak | 0.5 | 500 | Yes | 0.771 | 0.837 | 0.867 | 0.886 | 0.900 |
| weak | 0.5 | 1,000 | No | 0.958 | 0.985 | 0.991 | 0.995 | 0.996 |
| weak | 0.5 | 1,000 | Yes | 0.772 | 0.828 | 0.854 | 0.872 | 0.883 |
| weak | 0.7 | 100 | No | 0.966 | 0.987 | 0.991 | 0.992 | 0.976 |
| weak | 0.7 | 100 | Yes | 0.793 | 0.872 | 0.913 | 0.937 | 0.946 |
| weak | 0.7 | 250 | No | 0.954 | 0.988 | 0.993 | 0.996 | 0.999 |
| weak | 0.7 | 250 | Yes | 0.654 | 0.739 | 0.783 | 0.808 | 0.831 |
| weak | 0.7 | 500 | No | 0.955 | 0.985 | 0.994 | 0.997 | 0.997 |
| weak | 0.7 | 500 | Yes | 0.635 | 0.712 | 0.752 | 0.778 | 0.796 |
| weak | 0.7 | 1,000 | No | 0.953 | 0.985 | 0.990 | 0.994 | 0.997 |
| weak | 0.7 | 1,000 | Yes | 0.629 | 0.693 | 0.720 | 0.743 | 0.756 |
| strong | 0.3 | 100 | No | 0.969 | 0.991 | 0.998 | 0.999 | 1.000 |
| strong | 0.3 | 100 | Yes | 0.908 | 0.956 | 0.975 | 0.984 | 0.988 |
| strong | 0.3 | 250 | No | 0.957 | 0.990 | 0.997 | 0.999 | 0.999 |
| strong | 0.3 | 250 | Yes | 0.837 | 0.908 | 0.935 | 0.952 | 0.962 |
| strong | 0.3 | 500 | No | 0.953 | 0.987 | 0.994 | 0.997 | 0.998 |
| strong | 0.3 | 500 | Yes | 0.812 | 0.883 | 0.910 | 0.929 | 0.939 |
| strong | 0.3 | 1,000 | No | 0.954 | 0.985 | 0.994 | 0.996 | 0.997 |
| strong | 0.3 | 1,000 | Yes | 0.808 | 0.870 | 0.893 | 0.909 | 0.921 |
| strong | 0.5 | 100 | No | 0.970 | 0.989 | 0.996 | 0.999 | 0.999 |
| strong | 0.5 | 100 | Yes | 0.850 | 0.916 | 0.948 | 0.962 | 0.971 |
| strong | 0.5 | 250 | No | 0.955 | 0.989 | 0.996 | 0.998 | 1.000 |
| strong | 0.5 | 250 | Yes | 0.729 | 0.818 | 0.861 | 0.884 | 0.901 |
| strong | 0.5 | 500 | No | 0.947 | 0.984 | 0.991 | 0.996 | 0.998 |
| strong | 0.5 | 500 | Yes | 0.697 | 0.781 | 0.815 | 0.842 | 0.864 |
| strong | 0.5 | 1,000 | No | 0.954 | 0.986 | 0.993 | 0.994 | 0.997 |
| strong | 0.5 | 1,000 | Yes | 0.673 | 0.752 | 0.790 | 0.816 | 0.830 |
| strong | 0.7 | 100 | No | 0.966 | 0.988 | 0.994 | 0.993 | 0.980 |
| strong | 0.7 | 100 | Yes | 0.778 | 0.875 | 0.918 | 0.941 | 0.949 |
| strong | 0.7 | 250 | No | 0.959 | 0.989 | 0.994 | 0.997 | 0.999 |
| strong | 0.7 | 250 | Yes | 0.611 | 0.711 | 0.757 | 0.795 | 0.833 |
| strong | 0.7 | 500 | No | 0.953 | 0.987 | 0.995 | 0.997 | 0.999 |
| strong | 0.7 | 500 | Yes | 0.573 | 0.664 | 0.716 | 0.747 | 0.772 |
| strong | 0.7 | 1,000 | No | 0.955 | 0.984 | 0.991 | 0.994 | 0.998 |
| strong | 0.7 | 1,000 | Yes | 0.548 | 0.620 | 0.658 | 0.688 | 0.710 |

Table S20. Nominal coverage of the 95% CIs in simulations obtained using the Abadie-Imbens standard error from the secondary set of simulations. From left to right the columns report the treatment assignment mechanisms, the proportions of treated subjects, the sample sizes, whether matching was carried out with or without replacement, and the levels of oversampling.

| ***Treatment assignment mechanism*** | ***Proportion of treated*** | ***Sample size*** | ***Oversampling=1*** | ***Oversampling=2*** | ***Oversampling=3*** | ***Oversampling=4*** | ***Oversampling=5*** |
| --- | --- | --- | --- | --- | --- | --- | --- |
| weak | 0.3 | 100 | 0.938 | 0.914 | 0.881 | 0.847 | 0.810 |
| weak | 0.3 | 250 | 0.935 | 0.926 | 0.918 | 0.909 | 0.902 |
| weak | 0.3 | 500 | 0.934 | 0.936 | 0.936 | 0.934 | 0.929 |
| weak | 0.3 | 1,000 | 0.946 | 0.946 | 0.948 | 0.949 | 0.944 |
| weak | 0.5 | 100 | 0.920 | 0.889 | 0.835 | 0.780 | 0.709 |
| weak | 0.5 | 250 | 0.921 | 0.912 | 0.903 | 0.886 | 0.872 |
| weak | 0.5 | 500 | 0.931 | 0.932 | 0.929 | 0.926 | 0.920 |
| weak | 0.5 | 1,000 | 0.940 | 0.943 | 0.942 | 0.940 | 0.939 |
| weak | 0.7 | 100 | 0.866 | 0.805 | 0.704 | 0.588 | 0.473 |
| weak | 0.7 | 250 | 0.897 | 0.878 | 0.852 | 0.824 | 0.791 |
| weak | 0.7 | 500 | 0.914 | 0.913 | 0.910 | 0.899 | 0.889 |
| weak | 0.7 | 1,000 | 0.924 | 0.925 | 0.920 | 0.918 | 0.917 |
| strong | 0.3 | 100 | 0.924 | 0.896 | 0.858 | 0.816 | 0.767 |
| strong | 0.3 | 250 | 0.925 | 0.905 | 0.894 | 0.879 | 0.861 |
| strong | 0.3 | 500 | 0.919 | 0.920 | 0.918 | 0.918 | 0.913 |
| strong | 0.3 | 1,000 | 0.927 | 0.933 | 0.931 | 0.930 | 0.927 |
| strong | 0.5 | 100 | 0.881 | 0.830 | 0.776 | 0.712 | 0.654 |
| strong | 0.5 | 250 | 0.890 | 0.880 | 0.867 | 0.849 | 0.829 |
| strong | 0.5 | 500 | 0.899 | 0.906 | 0.896 | 0.891 | 0.883 |
| strong | 0.5 | 1,000 | 0.918 | 0.921 | 0.921 | 0.916 | 0.913 |
| strong | 0.7 | 100 | 0.816 | 0.754 | 0.662 | 0.557 | 0.459 |
| strong | 0.7 | 250 | 0.852 | 0.827 | 0.797 | 0.759 | 0.726 |
| strong | 0.7 | 500 | 0.876 | 0.881 | 0.872 | 0.860 | 0.844 |
| strong | 0.7 | 1,000 | 0.901 | 0.909 | 0.904 | 0.898 | 0.892 |


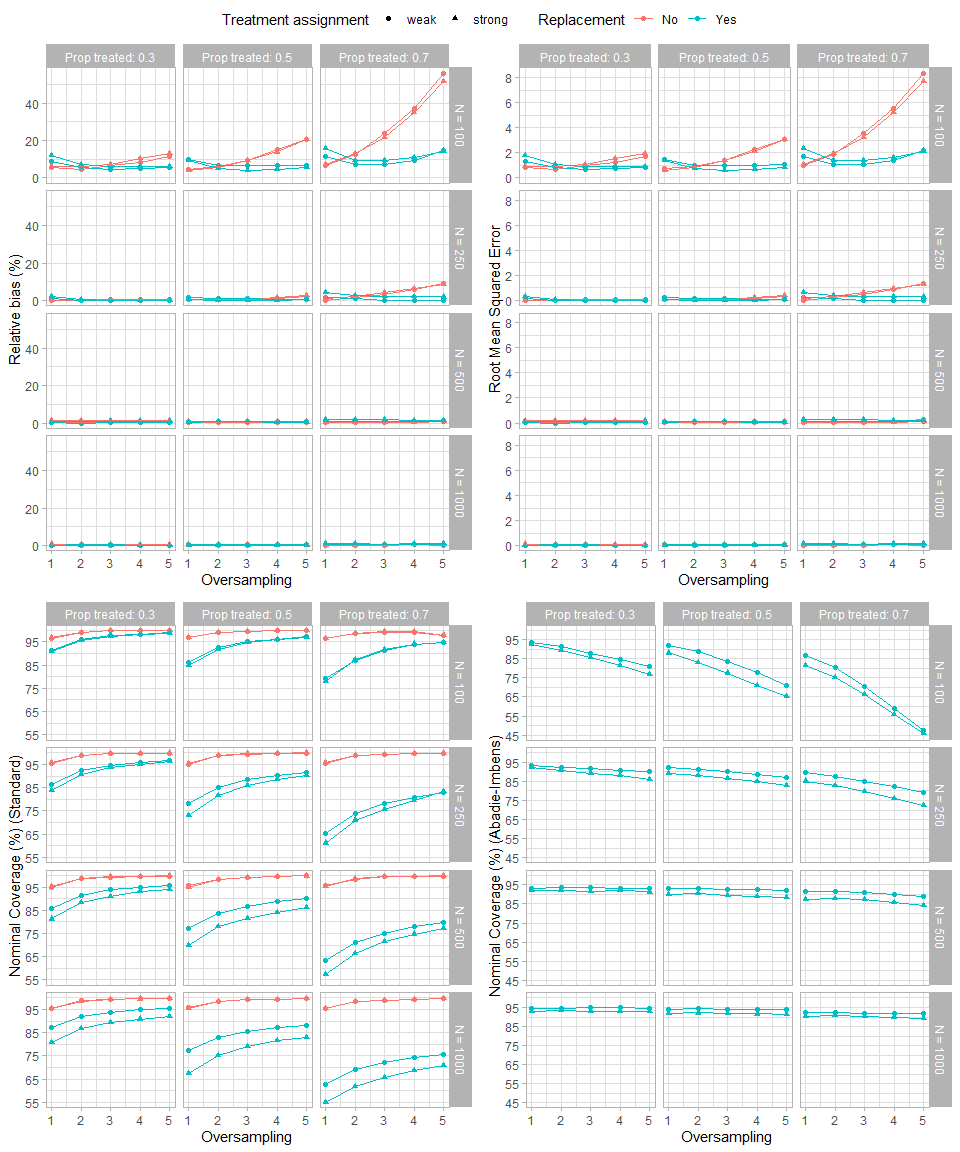


*Figure S1. Performances of the Average Treatment effect on the Treated (ATT) estimator on the matched sets obtained in each scenario from the secondary set of Monte Carlo simulations. The top-left plot shows the relative bias, whereas the Root Mean Squared Error (RMSE) is shown in the top-right plot. On the bottom-left side, the 95% Nominal Coverage (NC) obtained with the standard method is depicted, whereas on the bottom-right side the 95% NC obtained with the Abadie-Imbens (AI) method is shown. On the x-axis, the level of oversampling is represented. Matching without and with replacement are identified by the colors. The shape of the dots distinguishes between weak and strong treatment assignment. The columns of the panel grids show the proportion of treated subjects in the dataset, whereas the rows show the sample size of the dataset.*
